# High‐κ Perovskite‐Like Ternary Niobium Oxide Dielectrics for 2D Electronics

**DOI:** 10.1002/adma.202520423

**Published:** 2026-01-08

**Authors:** Biao Zhang, Jianmiao Guo, Jianmin Yan, Jialiang Wang, Chao Yun, Guang Zeng, Jie Li, Cong Wang, Zhengdao Xie, Yanglong Hou, Yang Chai

**Affiliations:** ^1^ Department of Applied Physics The Hong Kong Polytechnic University Hong Kong China; ^2^ Joint Research Center of Microelectronics The Hong Kong Polytechnic University Hong Kong China; ^3^ School of Materials Shenzhen Campus of Sun Yat‐Sen University Shenzhen China

**Keywords:** 2D, field‐effect transistors, high‐κ, ternary niobium oxides

## Abstract

High‐κ dielectrics with exceptional interface quality are essential for the field‐effect control of nanoscale transistors. However, their design remains challenging due to competing atomic‐scale polarization requirements. Here, we demonstrate nonlayered perovskite‐like ternary niobium oxides (CaNb_2_O_6_, KNb_3_O_8_, and Na_2_Nb_4_O_11_) as promising candidates, where strong Nb 4*d*‐O 2*p* covalent hybridization enables pronounced Nb^5+^ ionic displacements and enhanced polarization, while ionic bonding from intercalated Ca/K/Na suppresses electronic transitions, widening the bandgap and enhancing stability via configurational entropy. We successfully synthesize these high‐quality nanoflakes through a scalable molten‐salt method. Crucially, these oxides demonstrate a combination of high dielectric constants (∼16, 9, and 68 for CaNb_2_O_6_, KNb_3_O_8_, and Na_2_Nb_4_O_11_, respectively), wide bandgaps (∼4 eV), large breakdown field strengths (> 4.9 MV cm^−1^), and excellent air stability. Furthermore, due to the low‐contamination transfer via a fully dry process, MoS_2_ field‐effect transistors with these gate dielectrics achieve low subthreshold swings (∼60 mV dec^−1^), ON/OFF ratios > 10^7^, gate leakage currents below 10^−6^ A cm^−2^, and ultralow trap densities. We show high‐performance NOT and NAND gates using a CaNb_2_O_6_ dielectric layer, with the inverter achieving a static power consumption of < 0.02 µW and a gain of ∼20. This work provides new opportunities for the development of next‐generation 2D electronics devices.

## Graphical abstract

High‐κ dielectrics are vital for scaled electronics. Here, a family of 2D high‐κ perovskite‐like ternary niobium oxides is synthesized via a molten salt‐assisted method. Their integration into FETs and logic gates reveals superior switching characteristics, providing a fresh material platform and new insights for the advancement of high‐performance 2D electronics.

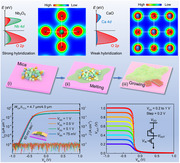

## 1 Introduction

Designing high‐performance dielectric materials through structural engineering remains a significant challenge due to the complex interplay between atomic‐scale characteristics (ionic radii, electron configurations, and orbital hybridization) and crystalline structure (crystal symmetry, coordination environments, and lattice dynamics) [1]. The dielectric constant (κ), which quantifies charge displacement polarization under an electric field, originates primarily from bound charge displacement (ionic and electronic contributions). Since electronic polarization typically contributes minimally to κ at operational frequencies, maximizing κ necessitates optimizing ionic separation while maintaining macroscopic charge neutrality [2, 3]. Concurrently, the rational design of dielectric materials demands careful optimization of electronic and crystal structures to simultaneously achieve high κ values, wide bandgaps, and excellent stability, which presents a significant challenge due to the fundamental trade‐offs. Metal oxides with stable electronic configurations often exhibit strong ionic dielectric responses [4, 5, 6]. Among these, perovskite high‐κ dielectric materials exhibit superior performance compared with binary oxides in terms of tunable dielectric properties and intrinsic defect suppression [7, 8, 9]. For instance, SrTiO_3_ achieves a significantly high κ (∼250), primarily attributed to soft‐mode distortions within the TiO_6_ octahedra of its perovskite structure [10, 11, 12]. The giant dielectric response stems from the strong covalency of Ti‐O bonds, driven by strong hybridization between Ti 3*d* and O 2*p* orbitals [10, 13, 14, 15, 16, 17]. The resultant anomalously large Born effective charge, which governs the strength of the ionic contribution to the macroscopic dielectric response, reflects the high efficiency of atomic displacements in generating polarization [16]. While recent advances in heterostructure engineering have successfully addressed the trade‐offs between maximum and remnant polarization in ferroelectrics [18], the relatively narrow bandgaps (3.0‐3.4 eV) of such perovskites (such as SrTiO_3_, BaTiO_3_) limit their application as dielectric layers [19].

In addition, conventional high‐κ materials prepared by in situ atomic‐layer deposition typically form amorphous phases with high defect densities, posing a risk of damage to underlying 2D semiconductors, limiting device performance [20]. In contrast, van der Waals (vdW) integration of high‐κ freestanding crystalline dielectrics with 2D semiconductors offers a damage‐free stacking process, enabling the formation of high‐quality dielectric/channel heterointerfaces [21, 22]. Therefore, designing suitable dielectric materials and controllably synthesizing freestanding 2D insulators are crucial for advancing 2D electronics.

In this work, we strategically identify three perovskite‐like ternary niobium oxides, namely CaNb_2_O_6_ (CNO), KNb_3_O_8_ (KNO), and Na_2_Nb_4_O_11_ (NNO). We synthesized these nanoflakes via liquid‐phase or vapor‐phase processes assisted by eutectic systems. The as‐grown crystals exhibit exceptional ambient stability, wide bandgaps, large dielectric constants, and high breakdown field strengths. By integrating these high‐κ dielectrics with MoS_2_ channels via a dry‐transfer process, the field‐effect transistors (FET) show high performance, including ON/OFF ratios over 10^7^, leakage currents <10^−6^ A cm^−2^, and near‐ideal SS of ∼60 mV dec^−1^, meeting the requirements of the 2022 International Roadmap for Devices and Systems for low‐power transistors [23]. Furthermore, we demonstrate functional logic gates (inverter and NAND) based on integrated CNO/MoS_2_ FETs, where the inverter operates at < 0.02 µW static power with a voltage gain of ∼20.

## 2 Results and Discussion

### 2.1 Theory of Ternary Niobium Oxides

**FIGURE 1:**
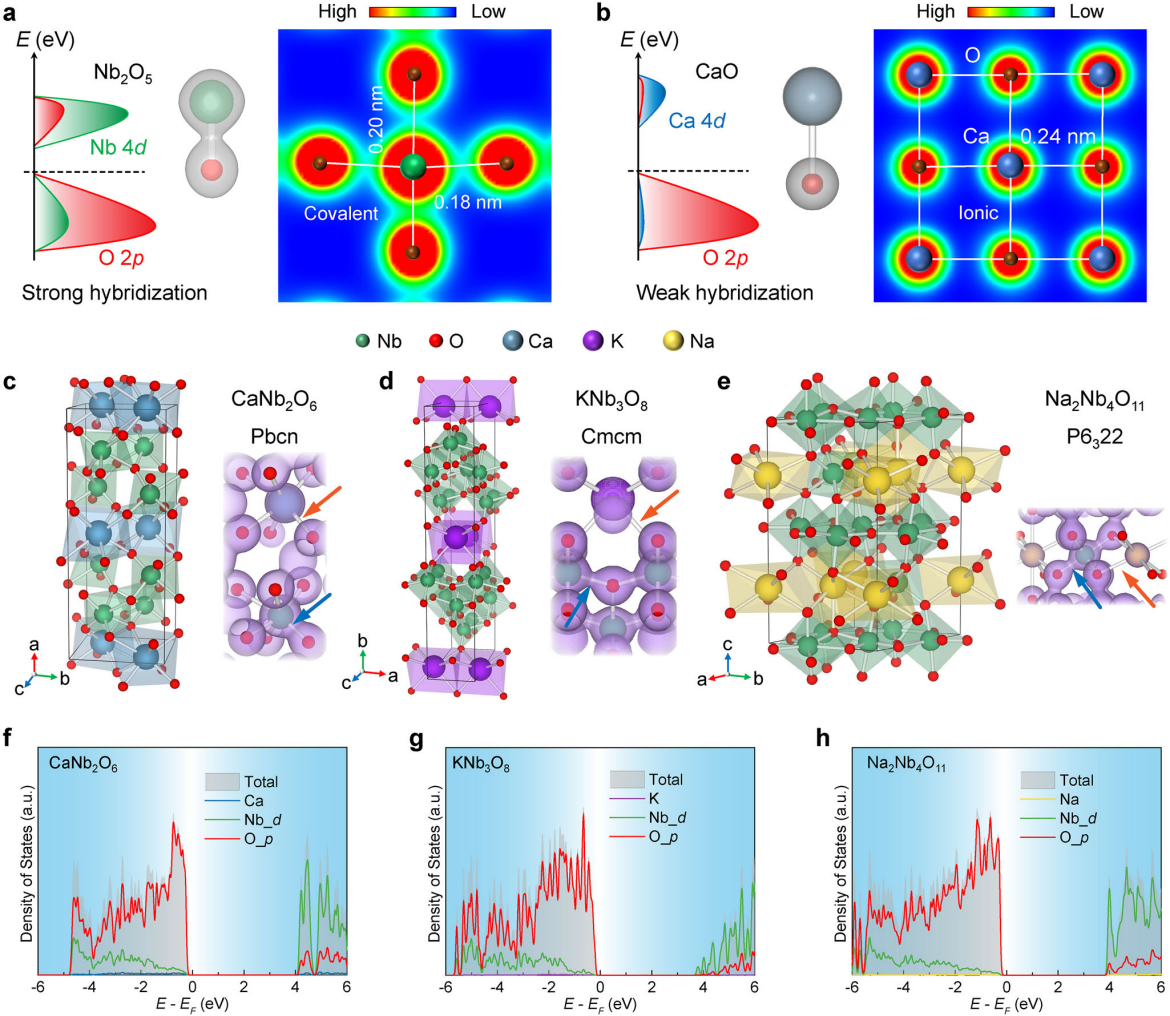
Theory of high‐κ perovskite‐like ternary niobium oxides. a) Nb_2_O_5_ band composition and charge density distribution on the Nb‐O plane (2D contour), demonstrating covalent Nb‐O bonding. b) Band composition of CaO and electronic charge density distribution on the Ca‐O plane, indicative of ionic Ca‐O bonding. c–e) Crystal structure information and corresponding charge densities (isosurfaces: 0.09 e/Bohr^3^) for the ternary niobium oxides. The blue arrows indicate the hybridized covalent bonds between Nb and O, and the orange arrows indicate the ionic bonds between Ca/K/Na and O. f–h) Calculated DOS of CNO (f), KNO (g), and NNO (h).

Our investigation of ternary niobium oxides begins with analyzing oxides with distinct electronic configurations. In Nb_2_O_5_, the *d*° configuration of Nb^5+^ promotes partial hybridization between Nb 4*d* and O 2*p* orbitals, resulting in strong covalent bonding between Nb and O (Figure 1a). In contrast, alkali and alkaline earth metals such as Na (Pauling electronegativity χ = 0.93), K (χ = 0.82), and Ca (χ = 1.01), which possess significantly lower electronegativities compared to Nb (χ = 1.59), exhibit weak hybridization (Figure 1b, as exemplified by CaO) with the highly electronegative oxygen (χ = 3.44), resulting in predominantly ionic bonding [24]. These stable valence states and electronic configurations preserve the fundamental bonding character within their ternary oxides. Figure 1c–e and Figure S1 present schematic crystal structures of CNO, KNO, and NNO. CNO and KNO crystallize in orthorhombic symmetry with lattice parameters *a* = 1.496 nm, *b* = 0.575 nm, *c* = 0.520 nm for CNO, *a* = 0.890 nm, *b* = 2.116 nm, *c* = 0.380 nm for KNO, while NNO adopts hexagonal symmetry with *a* = 0.625 nm, *c* = 1.237 nm [25, 26, 27, 28]. Despite structural distinctions, all three nonlayered materials feature interconnected NbO_6_ octahedra or NbO_7_ pentagonal bipyramids via edge‐/vertex‐sharing networks, with Ca^2+^, K^+^, or Na^+^ ions intercalated between or inside layers. The perovskite‐like structure provides space and elastic flexibility for Nb^5+^ displacement, facilitating the polarization under an electric field. Furthermore, the covalent bonds between Nb and O orbitals observed from the charge density (indicated by blue arrows) enhance the lattice's capacity for ionic displacement polarization [13, 15]. Moreover, the strong ionic bonds (indicated by orange arrows) between Ca/Na/K and O reduce the probability of electron transition between Nb‐*d* and O‐*p* and consequently widen the bandgaps.

The density of states (DOS) of these three structures using density functional theory was also calculated (Figure 1f–h), demonstrating dominant contributions from O‐2*p* orbitals in the valence band and Nb‐4*d* orbitals in the conduction band with *d*‐*p* hybridization. This orbital mixing enhances Nb‐O covalency while critically reducing bond rigidity. The softened lattice exhibits diminished resistance to relative Nb^5+^/O^2−^ displacements under electric fields, enabling larger ionic shifts which trigger amplified polarization [16]. The Ca^2+^, K^+^, and Na^+^ ions adopt closed‐shell electronic configurations, exhibiting negligible contributions to both the valence and conduction bands near the Fermi level. However, the energy levels of O and Nb orbitals can be indirectly affected by the crystal field, thus increasing the band gap compared to that of Nb_2_O_5_ (∼3.2 eV) [29]. Simultaneously, these intercalated ions introduce configurational entropy into the lattice, enhancing chemical and thermodynamic stability. The strong ionic bonds and the oxygen polyhedral coordination also create a high migration energy barrier for Ca/Na/K ions, which ensures the structural and electrical stability. Therefore, these characteristics enable a favorable balance of high dielectric constants, wide bandgaps, and robust stability in these materials.

### 2.2 Synthesis and Structure Characterizations

**FIGURE 2:**
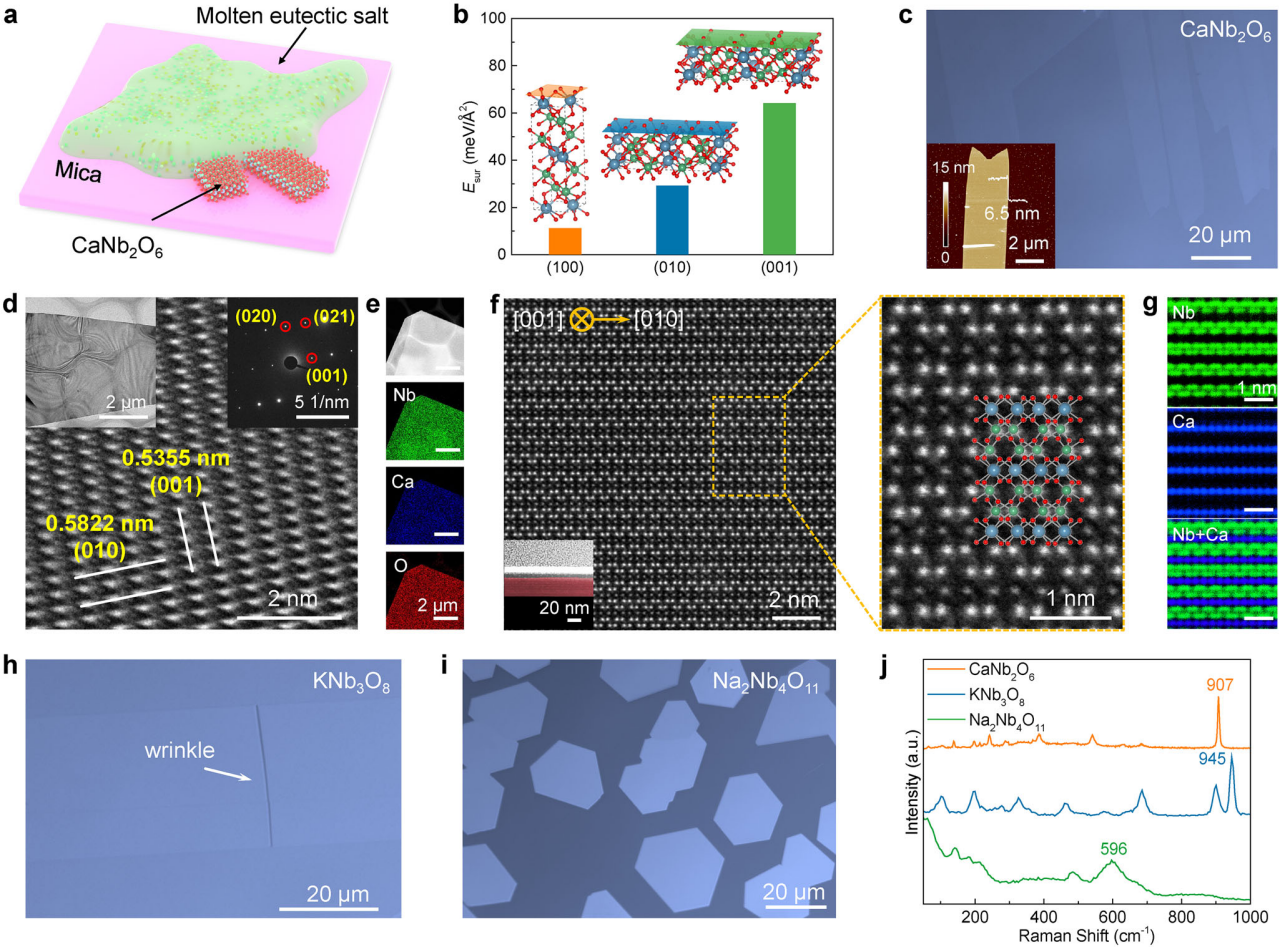
Synthesis, growth mechanism, and characterizations. a) Schematic view of the growth mechanism of CNO crystals. b) The calculated *E*_sur_ of (100), (010), and (001) planes of CNO. c) Optical image of the as‐grown CNO nanoflakes. The inset is the AFM image of a CNO nanoflake with a thickness of ∼6.5 nm. d) HRTEM image of a CNO nanoflake. The insets are the corresponding low‐magnification bright‐field transmission electron microscopy (TEM) image (top left) and the SAED pattern (top right). e) The high‐angle annular dark field (HAADF) TEM image and corresponding elemental mappings of Nb, Ca, and O, respectively. f) Cross‐sectional HAADF‐STEM images of the CNO (001) plane and the high‐magnification image. g) The atomic‐resolution EDS mappings of Nb and Ca. h, i) Optical images of the as‐synthesized KNO (h) and NNO (i) nanoflakes. j) Raman spectra of CNO, KNO, and NNO nanoflakes.

Initially, growing 2D CNO via standard chemical vapor deposition (CVD) processes resulted in excessively high nucleation densities, yielding only small crystalline domains with limited lateral dimensions (Figure S2). To overcome the limitations, we employed a substrate‐assisted molten salt growth strategy [30]. As illustrated in Figure 2a and Figure S3, the growth process employed mica as the substrate with Nb_2_O_5_ and CaCl_2_·H_2_O as precursors, along with KCl‐LiCl eutectic salt (41.8 mol% KCl). The eutectic system reduces the growth temperature by providing a liquid‐phase environment [31]. At the same time, the added CaCl_2_ can further form a ternary eutectic salt to lower the melting point (Figure S4) [32]. The liquid molten salt facilitates enhanced dissolution of chemical precursors, accelerates ion diffusion rates, lowers the reaction threshold, and promotes the assembly of fundamental building blocks [33]. Specifically, as the temperature rises, the ions (Ca^2+^, K^+^, Li^+^, Cl^−^, and chloroxyniobates/niobium‐oxo anions) dissolve into the eutectic melt, forming a homogeneous solution. Herein, Cl^−^ ions facilitate the dissolution of Nb_2_O_5_ by forming soluble chloroniobate ions [34]. Once the reaction temperature is reached, CNO nucleation initiates at the atomic scale along the solution boundary. Then the growth is governed by the thermodynamics of anisotropic surface energy and the kinetics of mass transport, promoting lateral growth along the atomically flat mica surface (Figure S5). Throughout this process, the molten salt medium provides a continuous and rapid supply of reactants. Furthermore, the most thermodynamically stable surface was analyzed through density functional theory (DFT) calculations. The (100) crystal plane of CNO exhibits the lowest surface energy (*E*_sur_) of 11.23 meV Å^−2^, compared to 29.20 and 64.09 meV Å^−2^ for the (010) and (001) planes, respectively (Figure 2b). Based on the liquid molten salt growth mechanism and the anisotropic surface energy of CNO, its 2D anisotropic growth is preferentially activated at the solution edge, resulting in maximum domain sizes exceeding 200 µm (Figure 2c and Figure S5), which is larger than that of most of the nonlayered 2D materials grown by salt‐assisted methods.

Meanwhile, an unconventional growth mechanism was revealed during the synthesis process (Figure S6), characterized by central nucleation followed by random orientation growth. This phenomenon differs fundamentally from conventional CVD growth processes and arises from the formation of small molten salt droplets during the reaction. Notably, the central nucleation region tends to exhibit amorphous characteristics, in contrast to the crystalline domains growing outward. In addition, the molten salt‐assisted synthesis demonstrates remarkable substrate versatility. We have successfully obtained CNO nanoflakes under identical growth conditions on a sapphire substrate, which possesses dangling bonds on the surface (Figure S7), indicating the universality of the method. The atomic force microscopy (AFM) image of CNO is shown in Figure 2c. The thickness of a nanoflake on mica is approximately 6.5 nm. Furthermore, some wrinkles can be observed on the as‐synthesized samples, demonstrating weak interfacial adhesion between the CNO layers and the substrate, suggesting that the nanoflakes can be readily transferred [2, 35, 36].

The composition and structure of CNO were systematically characterized. X‐ray photoelectron spectroscopy (XPS) analysis verified the exclusive presence of Ca^2+^ and Nb^5+^ oxidation states (Figure S8) [27]. High‐resolution transmission electron microscopy (HRTEM) imaging confirmed the single‐crystalline nature of the nanoflakes, with measured interplanar spacings of 0.5355 nm (*d*_001_) and 0.5822 nm (*d*_010_), corresponding to the (100) crystallographic planes (Figure 2d). Besides, rectangular diffraction symmetry is observed in the selected‐area electron diffraction (SAED) pattern aligned with the [100] orientation. In addition, elemental mapping demonstrated homogeneous spatial distribution of constituent elements with a stoichiometric ratio of Ca: Nb: O ≈ 1:2:6 (Figure 2e and Figure S9). To investigate the out‐of‐plane atomic structure, cross‐sectional samples were prepared by focused‐ion beam (FIB) milling along the short edge (Figure S10). Energy‐dispersive X‐ray (EDS) spectroscopy mapping revealed the uniform distribution of Ca, Nb, and O over the entire sample (Figure S10e–h). Atomic‐resolution characterization via high‐angle annular dark‐field scanning transmission electron microscopy (HAADF‐STEM) revealed well‐ordered atomic arrangements in the (001) plane (Figure 2f). The *Z*‐contrast mechanism enabled clear differentiation between Ca (*Z* = 20) and Nb (*Z* = 41) atomic rows based on their distinct intensity, consistent with the crystal structure. Furthermore, the atomic‐resolution EDS spectroscopy mapping provided direct visualization of the in‐plane cation distribution (Figure 2g). Electron energy‐loss spectroscopy (EELS) was also performed to confirm the chemical composition (Figure S11).

Furthermore, we successfully synthesized KNO and NNO nanoflakes using a CVD approach with KCl (for KNO) [25] or KCl‐NaCl (for NNO) molten salts [37] (Figure S12). The minimum thicknesses of KNO and NNO nanoflakes are 3.4 and 9.7 nm. However, liquid‐phase synthesis of KNO and NNO nanoflakes at lower temperatures typically results in poor dimensional and morphological control (Figure S13). In contrast, KNO and NNO nanoflakes can achieve large domain sizes through a vapor‐phase growth mechanism. The difference in growth behavior can be attributed to the higher reaction temperature used in the CVD process. The elevated temperature enhances atomic mobility, thereby reducing the nucleation density and promoting diffusion of adatoms [34]. Although CNO and KNO can be synthesized by lower‐temperature chemical methods, such as hydrothermal and sol‐gel methods [38, 39], the resulting limited nanoscale dimensions and poor crystallinity significantly hinder their practical application in electronic devices. Figure 2h,i shows the optical images of KNO and NNO nanoflakes. It is worth noting that the KNO surface also has obvious wrinkles, indicating a weak adhesion force with the substrate. HRTEM and EDS analyses further corroborate the structural integrity and chemical composition of both KNO and NNO nanoflakes (Figures S14 and S15). Notably, the microstructure and chemical composition of NNO were further determined by HAADF‐STEM characterization (Figure S16). In addition, DFT calculations of the surface energies confirm that the (010) plane of KNO and the (001) plane of NNO are thermodynamically stable, in agreement with the experimental results (Figure S17). The most stable surfaces for all three crystals are parallel to the Nb‐O polyhedral layers, consistent with the intercalation structure. The energy difference between the most stable and the second‑most stable surface is smaller for NNO (36.3%) than for CNO (61.5%) or KNO (77.1%). This indicates that NNO grows more readily in the direction perpendicular to its most stable plane, resulting in thicker nanoflakes compared to CNO and KNO. Furthermore, statistical analysis of the maximum lateral size and minimum thickness for all three materials was conducted (Figure S18), indicating their reproducibility and scalability.

Raman spectroscopy analysis (Figure 2j) revealed distinct vibrational properties among the three materials. Both CNO and KNO exhibited strong Raman signals with peak positions and intensities matching previous reports, proving their crystal phases [25, 27]. In comparison, NNO displayed relatively weaker Raman activity, which may be due to the different phonon scattering properties. Furthermore, all three materials demonstrate exceptional atmospheric stability, as evidenced by the absence of significant degradation in both Raman spectral features and surface characteristics after 6 months of ambient air exposure (Figures S19 and S20).

### 2.3 Dielectric Property Characterization of CNO, KNO, and NNO

**FIGURE 3:**
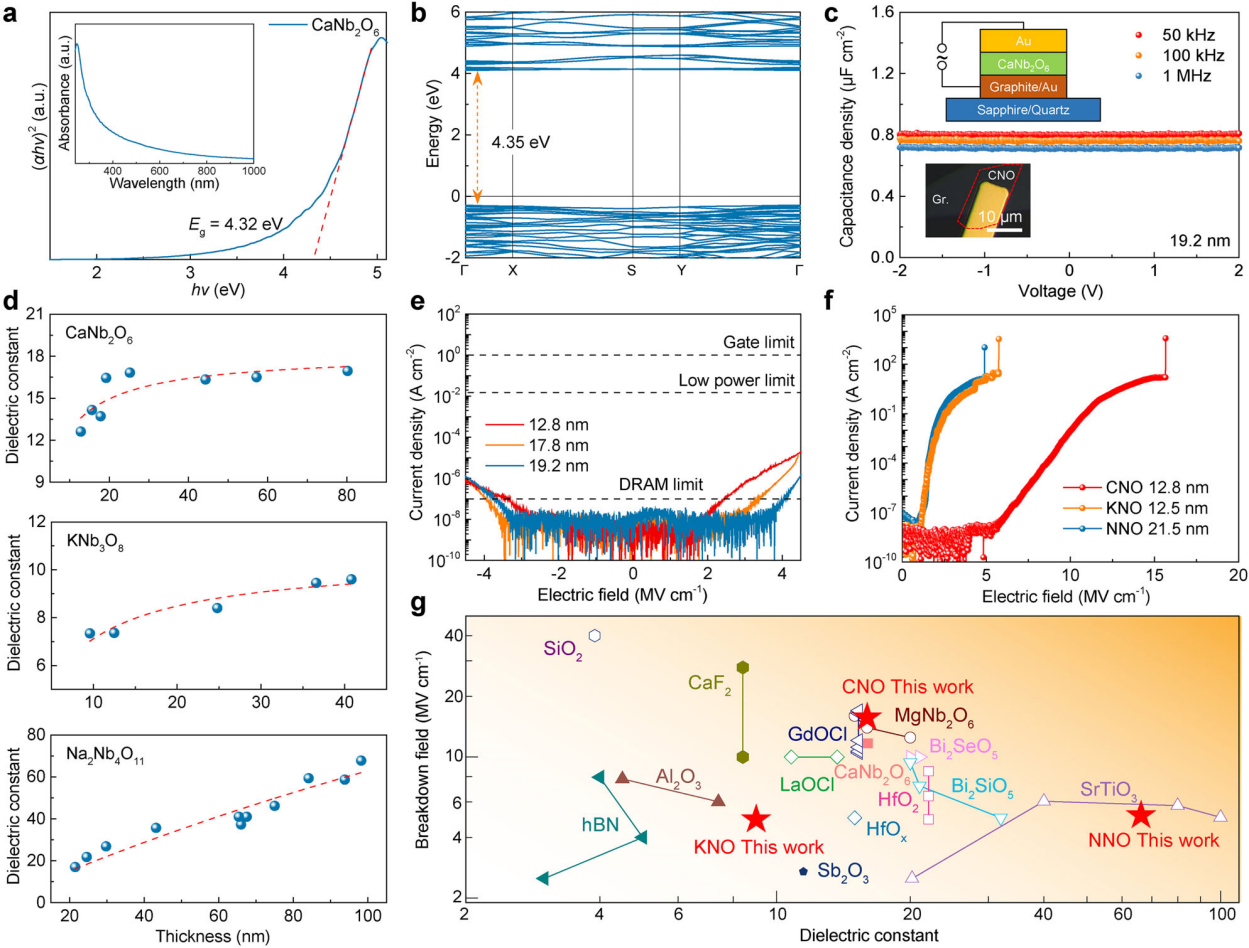
Dielectric properties of CNO, KNO, and NNO. a) Optical bandgap determination of CNO nanoflake via Tauc plot analysis, *α*, *h*, and *ν* represent the absorption coefficient, Planck constant, and light frequency, respectively. Inset: corresponding micro‐area UV–vis absorption spectrum. b) Calculated electronic band structure of 2D CNO using HSE06 functional. c) *C*–*V* characteristics of a 19.2‐nm‐thick CNO MIM device. Insets: OM image (bottom) and schematic illustration (top) of the MIM device structure. d) Thickness‐dependent dielectric constants of CNO, KNO, and NNO nanoflakes measured by *C*‐*V* analysis. e) Thickness‐dependent leakage current density of CNO nanoflakes under varying electric fields. The dashed lines indicate the limits for relevant applications. f) Breakdown characteristics of CNO, KNO, and NNO nanoflakes. g) *E*_BD_ and effective dielectric constant of CNO, KNO, and NNO nanoflakes, compared with other dielectric materials.

Following the successful growth of the ternary oxides, we systematically investigated their dielectric properties. The bandgap of crystalline CNO was determined via micro‐area ultraviolet‐visible (UV–vis) absorption spectroscopy (Figure 3a), yielding a value of 4.32 eV, which is consistent with the bandgap of bulk crystal and the nanoflakes synthesized by CVD [28, 40]. DFT calculations using the HSE06 functional indicate a direct bandgap insulator with a gap of 4.35 eV (Figure 3b), in agreement with previous reports [27, 40, 41]. We also calculated the band structure of CNO using standard GGA and DFT + *U* methods (Figure S21). The bandgap linearly increases from 3.85 to 4.44 eV by scanning *U* from 0.5 to 5.5 eV. However, the standard GGA functional yields a smaller gap of 3.82 eV. This discrepancy arises from the well‐known DFT bandgap underestimation and parity‐forbidden transitions induced by the inversion symmetry in CNO crystals [25]. The calculated band structures of KNO and NNO crystals exhibit indirect bandgaps of 3.98 and 4.12 eV, respectively (Figure S22). Similarly, the optical bandgaps of KNO and NNO nanoflakes were characterized, as shown in Figure S23, exhibiting bandgaps of 4.05 and 3.90 eV, respectively, close to theoretically predicted values. To verify the feasibility of these nanoflakes as gate dielectrics, capacitance‐voltage (*C*‐*V*) measurements were performed using parallel‐plate capacitor devices. Notably, quartz or sapphire insulating substrates were employed to minimize parasitic capacitance effects [42]. The device structure comprised either Au or graphite bottom electrodes paired with transferred Au top electrodes, both exhibiting excellent surface flatness to minimize capacitance measurement variations. Figure 3c displays the *C*‐*V* characteristics of a 19.2 nm‐thick CNO dielectric layer, demonstrating stability across a ± 2 V bias range without observable fluctuations. The relative dielectric constant (*ε*_r_) was determined according to the parallel‐plate capacitance model (Equation 1):

(1) $$C=\frac{A\;\varepsilon_{r}\varepsilon_{0}}{t_{\mathrm{ox}}}$$

where *C* represents measured capacitance, *A* the electrode overlap area, *t*_o__x_ the dielectric thickness, and *ε*_0_ the vacuum permittivity. As summarized in Figure 3d, the thickness‐dependent *ε*_r_ measured at 100 kHz for all three materials exhibits a consistent decreasing trend with reduced thickness, in agreement with the ‘dead layer’ effect where interfacial regions between electrodes and dielectric material demonstrate significantly reduced *ε*_r_ compared to the bulk material's intrinsic dielectric response [43]. The corresponding optical images of the devices and *C*‐*V* curves are presented in Figures S24–S29. The *ε*_r_ of CNO nanoflakes with thicknesses exceeding 20 nm reaches ∼16, much higher than that of SiO_2_ (3.9) [5] and 2D hexagonal boron nitride (*h*‐BN) (2‐4) [44]. KNO exhibits a more moderate *ε*_r_ of 9 at 40 nm, which is close to the bulk crystal [45]. Notably, NNO demonstrates exceptional *ε*_r_ reaching 36 (43.2 nm thickness) and 68 (98.2 nm thickness), outperforming conventional high‐*κ* dielectrics including HfO_2_ [4] and Al_2_O_3_ [46]. The higher *ε*_r_ of NNO compared to CNO and KNO can be attributed to its distinct structural configuration, where 82% of the Nb sites form NbO_7_ pentagonal bipyramids, in contrast to the typical NbO_6_ octahedra in CNO and KNO (Figure S1). The difference in the coordination polyhedron may enhance the lattice flexibility and local polarization capability of NNO. Within the looser environment, the transverse optical vibrational mode of the ions becomes more susceptible to low‐frequency excitation. The phonon mode softening enables a stronger ionic displacement polarization response to an applied electric field [10, 47]. The equivalent oxide thickness (EOT) of the dielectrics was also evaluated using the relation EOT = 3.9 *t*/*ε*_r_, where *t* is the minimum physical thickness of the MIM devices, and 3.9 is the dielectric constant of silicon oxide. The calculated EOT values for the devices are approximately 4.0 nm for CNO (*t* = 12.8 nm), 5.4 nm for KNO (*t* = 9.6 nm), and 4.9 nm for NNO (*t* = 21.5 nm). Although the EOTs are currently limited by these conservative physical thicknesses and the interfacial ‘dead layer’ effect, the intrinsic dielectric constants demonstrate potential for lower EOTs by further interface optimization and physical scaling. Another essential requirement for gate insulators is ultralow leakage current. For CNO samples with thicknesses ranging from 12.8 to 19.2 nm, the measured leakage current densities demonstrate exceptional performance (Figure 3e), remaining below 5 × 10^−8^ A cm^−2^ at an applied field of ± 2 MV cm^−1^ (surpassing DRAM requirements) and below 1 × 10^−4^ A cm^−2^ at 4.5 MV cm^−1^, which is significantly lower than both the low‐power operation limit (1.5 × 10^−2^ A cm^−2^) and standard complementary metal‐oxide‐semiconductor (CMOS) gate limit (10 A cm^−2^).

Through application of elevated current bias to the metal‐insulator‐metal (MIM) devices, we characterized the breakdown behavior of the three dielectric materials (Figure 3f). Attributed to its wide bandgap, excellent crystallinity, and the atomically smooth graphite electrode interface, the 12.8 nm CNO sample demonstrates exceptional breakdown field strength (*E*_BD_) of 15.7 MV cm^−1^, approximately three times greater than perovskite SrTiO_3_ membranes reported in previous studies [9]. Meanwhile, the *E*_BD_ of 12.5 nm KNO and 21.5 nm NNO is 5.8 and 4.9 MV cm^−1^, respectively. Figure 3g summarizes a comprehensive comparison of these materials against state‐of‐the‐art dielectrics employed in both silicon and 2D semiconductor technologies in two axes of dielectric constant and *E*_BD_ [4, 5, 9, 20, 28, 44, 48, 49, 50, 51, 52, 53, 54, 55, 56, 57, 58, 59]. The CNO, KNO, and NNO crystals demonstrate comparable properties among these materials. The key gate insulator parameters, such as bandgap, dielectric constant, and breakdown field strength, are listed in Table S1. These results demonstrate that the substantial bandgaps of the three ternary oxides not only enable their superior dielectric performance, manifested through ultralow leakage currents and high breakdown fields, but also establish a foundation for developing multifunctional applications in FET architectures. The combination of these properties suggests significant potential for next‐generation electronic devices requiring robust gate dielectrics with enhanced performance characteristics.

### 2.4 High‐Performance 2D Transistors with High‐*κ* Gate Dielectrics

**FIGURE 4:**
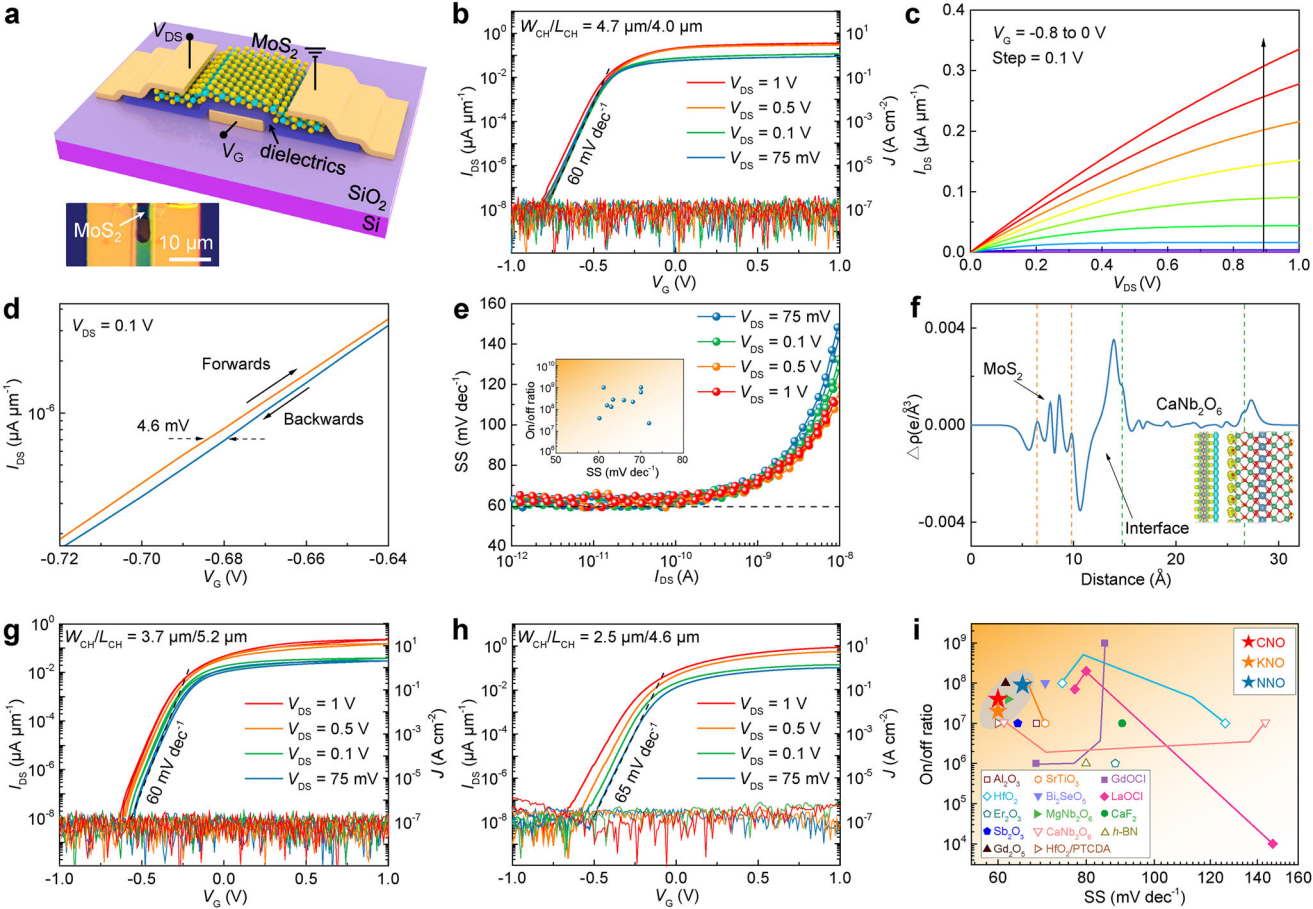
High‐performance 2D transistors incorporating high‐*κ* gate dielectrics. a) Schematic illustration of bottom‐gate MoS_2_ FET. b) Dual‐sweep transfer characteristics (*I*_DS_‐*V*_G_) of a MoS_2_ FET employing CNO gate dielectrics under varying *V*_DS_. c) Corresponding output characteristics (*I*_DS_‐*V*_DS_). d) The hysteresis width at *V*_DS_ = 0.1 V. The arrows indicate the forward and reverse voltage sweep directions. e) Extracted SS values versus *I*_D_ of the same device. Inset: Summary of SS and ON/OFF current ratio characteristics of 10 devices. f) Plane‐averaged differential charge density profile of the CNO/MoS_2_ vdW heterostructure along the *z*‐axis. The orange dashed line indicates the S boundary of MoS_2,_ and the blue dashed line marks the O atomic plane of CNO. Inset: Cross‐sectional views of the differential charge density distribution, where blue and yellow isosurfaces (0.00064 e/Bohr^3^) represent electron depletion and accumulation regions, respectively. g, h) Transfer curves of bottom‐gate MoS_2_ FETs utilizing the (g) KNO and (h) NNO gate dielectrics under various *V*_DS_. i) Performance comparison of state‐of‐the‐art MoS_2_ transistors with high‐κ dielectrics, evaluating SS and ON/OFF current ratios. All devices were measured at 300 K.

To evaluate gate dielectric performance, monocrystalline CNO, KNO, and NNO were integrated with exfoliated 2D MoS_2_ to fabricate bottom‐gated transistors. Remarkably, the weak interfacial interactions between CNO/KNO and mica substrates facilitated high‐quality exfoliation and well‐aligned transfer of the nanoflakes. For other thin‐layered insulators grown by CVD, the strong interactions with substrates inevitably pose challenges to clean transfer and subsequent vdW integration [52, 60]. The commonly used wet transfer methods, which involve deionized water or acidic/alkaline solution, often leave residual polymer, solvent molecules, or impurities on the surface of 2D materials. These contaminants can create defect states or trap sites within the 2D material, leading to increased gate leakage current and higher SS. Also, the charge trapping/detrapping at the interface can ultimately cause hysteresis in the FET's transfer characteristics, resulting in unreliable switching behavior and reduced device stability [61]. In contrast, the polydimethylsiloxane (PDMS)‐assisted dry transfer approach enables a cleaner interface by minimizing residual contaminants (Figure S30). Specifically, CNO and KNO nanoflakes are first adhered to PDMS at room temperature. Then the nanoflakes are precisely placed onto pre‐deposited Cr/Au electrodes on Si/SiO_2_ substrate. Subsequent heating softens the PDMS, enabling the release of the nanosheets, followed by sequential transfer of MoS_2_ channel material and source/drain electrodes, leading to an exceptionally clean interface between the dielectric, MoS_2_, and electrodes. Furthermore, the clean vdW gap serves as an effective tunneling barrier, significantly reducing carrier tunneling probability and thus suppressing gate leakage currents [21, 42]. The device architecture and the optical image are shown in Figure 4a. The fabricated device features a MoS_2_ layer with a thickness of 4.8 nm, a CNO dielectric of 29.5 nm, with channel width (*W*_CH_) and channel length (*L*_CH_) of 4.7 and 4.0 µm, respectively. The electrical characterization reveals outstanding device performance as shown in Figure 4b,c. The double‐sweep transfer curves (drain current vs gate voltage, *I*_DS_‐*V*_G_) display a typical *n*‐type behavior by applying a bottom gate voltage (*V*_G_) within the range of ±1 V. The transfer characteristics exhibit a high current ON/OFF ratio (*I*_on_/*I*_off_) up to 4 × 10^7^ and an ultralow leakage current density below 3 × 10^−7^ A cm^−2^, which has exceeded the detection limit of the instrument. There was a sharp increase in the subthreshold region with the SS value of ∼60 mV dec^−1^, approaching the Boltzmann thermionic limit at room temperature, indicating efficient gate control of the CNO nanoflake. The field‐effect mobility was calculated as 24.4 cm^2^ V^−1^ s^−1^ (Figure S31). Corresponding output characteristics presented in Figure 4c reveal linear *I*_DS_‐*V*_DS_ behavior at low bias voltages transitioning to saturation at higher biases, confirming the effective current modulation. The devices show negligible hysteresis with Δ*V*_G_ = 4.6 mV (Figure 4d), obtained from the enlarged view of the transfer curve at *V*_DS_ = 0.1 V and *I*_DS_ = 10^−6^ µA µm^−1^ with a slow sweeping rate of 0.05 V s^−1^, reflecting an atomically clean heterointerface between MoS_2_ and CNO dielectric with negligible interfacial defect density due to the dry transfer process with minimal residue. Furthermore, the CNO‐gated MoS_2_ FETs maintain consistently low SS values close to ∼60 mV dec^−^^1^ across varying drain currents from 10^−12^ to 10^−10^ A during both forward and reverse voltage sweeps, indicating the high quality of the CNO/MoS_2_ interfaces with low interfacial trap density (*D*_it_) (Figure 4e). The *D*_it_ was calculated using the following Equation 2:

(2) $$\mathrm{SS}\;=\;\ln\left(10\right)\frac{k_{B}T}{q}\left(1+\frac{qD_{\mathrm{it}}}{C_{G}}\right)$$

where *k*_B_ and *q* denote the Boltzmann constant (1.38 × 10^−23^ J/K) and elementary charge (1.6 × 10^−19^ C), respectively, and *C*_G_ represents the gate capacitance. The achieved ultralow *D*_it_ value of 2.24 × 10^10^ cm^−2^ eV^−1^ demonstrates superior performance compared to conventional 2D MoS_2_ FETs [2, 9, 42, 46, 52, 54, 59, 60, 62, 63, 64, 65]. To demonstrate device reproducibility, we fabricated a few additional bottom‐gate FETs using the same device fabrication process. All these devices exhibited performance with ON/OFF ratios ranging from 10^7^ to 10^9^, SS of approximately 60–70 mV dec^−1^, and hysteresis of less than 30 mV (Figure 4e and Figure S32). The performance variation originates primarily from the stochasticity in the device fabrication process, especially the variations in the thickness and shape of the exfoliated few‐layer MoS_2_ and CNO nanoflakes. In addition to bottom‐gate transistors, we also prepared three top‐gate FETs to investigate the performance. The transfer curves display slightly inferior performance due to less effective gate control caused by the non‐gated areas (Figure S33). Moreover, we calculated the interface interaction between MoS_2_ and CNO nanoflakes (Figure 4f). The planar‐averaged charge density difference profile along the surface‐normal direction reveals distinct interfacial boundaries. The gap between these two material systems averages 4.9 Å. This interfacial separation substantially exceeds the sum of the covalent radii of oxygen (0.73 Å) and sulfur (1.02 Å) atoms, providing further evidence for the formation of a vdW heterointerface.

In addition to CNO, the performance of KNO and NNO nanoflakes was also investigated as the gate dielectric layer of MoS_2_ based bottom‐gate FETs. Owing to the same dry transfer process, the KNO FET devices have high performance derived from the transfer curves in Figure 4g. The high ON/OFF ratio was 2 × 10^7^ and the leakage was below 3 × 10^−7^ A cm^−2^ while sweeping the *V*_G_ between −1.0 and 1.0 V. Furthermore, the drain current presents a sharp increase in the subthreshold region with SS value of ∼60 mV dec^−1^ (Figure 4g and Figure S34a), which approaches the theoretical limit at 300 K. Although the *ε*_r_ of KNO is ∼9, the resulting device performance remains excellent, indicating the high crystal quality and clean interfaces between MoS_2_ and KNO nanoflakes of the device. The plot of the corresponding output curve (Figure S34c) shows linear behavior at low bias and saturation at high bias. Meanwhile, the FET using NNO nanoflakes as the gate dielectric was also prepared to evaluate device performance. As shown in Figure 4h, the device exhibits characteristics including the high current on/off ratio up to 9 × 10^7^, ultralow leakage current density below 2 × 10^−6^ A cm^−2^, and the low SS value of ∼65 mV dec^−1^ (Figure 4g and Figure S34b). The field‐effect mobility for the KNO and NNO‐dielectric FETs is calculated as 14.5 and 18.0 cm^2^ V^−1^ s^−1^ at *V*_DS_ = 0.075 V within the subthreshold region. The output characteristics also show linear features when at small *V*_DS_ (Figure S34d). Furthermore, the *D*_it_ at the KNO/MoS_2_ and NNO/MoS_2_ heterointerfaces were also evaluated from Equation 2. An extremely low *D*_it_ approximately 9.4 × 10^9^ cm^−2^ eV^−1^ for KNO/MoS_2_ and 2.7 × 10^11^ cm^−2^ eV^−1^ for NNO/MoS_2_ can be obtained. Moreover, the band alignments further contribute to the key device metrics. DFT results indicate that the wide bandgaps of CNO, KNO, and NNO lead to band offsets greater than 1 eV relative to MoS_2_ for both the conduction band minimum and the valence band maximum (Figure S22e), thus enabling excellent gate control and leakage suppression. To confirm the reproducibility of performance metrics, we fabricated an additional device for each of KNO and NNO as bottom‐gate dielectrics, with each exhibiting reliable and consistent performance within its respective materials (Figure S35). As summarized in Figure 4i, the devices show competitive *I*_on_/*I*_off_ and SS values among the state‐of‐the‐art MoS_2_ transistors integrated with high‐κ dielectrics, particularly the extremely low SS [2, 9, 20, 28, 30, 42, 46, 51, 52, 53, 54, 55, 59, 60, 62, 63, 64, 65, 66, 67]. The comparison of the FETs, including SS, *I*_on_/*I*_off_, and *D*_it_, is listed in Table S2. The high performance of the transistors highlights the great potential of these ternary niobium oxides for application in 2D electronics.

### 2.5 Low‐Power 2D Logic Gates Based on CNO/MoS_2_ Transistors

**FIGURE 5:**
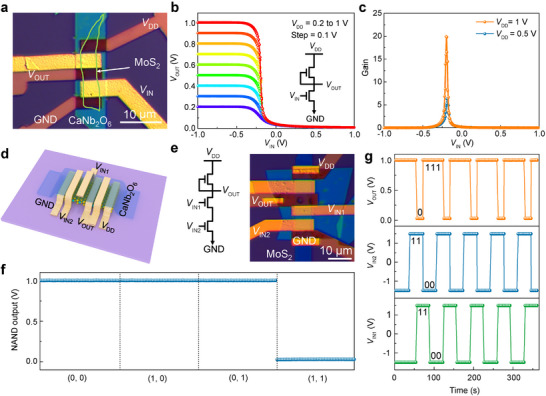
Logic gates implemented using CNO/MoS_2_ transistors. a) Optical image of an *n*‐type MOS inverter comprising two CNO/MoS_2_ FETs. b) Voltage transfer characteristics of the inverter, with an inset showing the corresponding circuit schematic. c) Derived voltage gain characteristics for the inverter shown in (b). d) Structure schematic of a NAND gate based on 2D CNO/MoS_2_ FETs. e) Circuit diagram and optical image of the fabricated NAND gate device. f) Input‐output logic functionality of the NAND gate at *V*_DD_ = 1 V. g) Dynamic output response of the NAND gate to a quasi‐static square‐wave input. GND denotes ground, *V*_IN_ the input voltage, *V*_OUT_ the output voltage, and *V*_DD_ the supply voltage.

The dry transfer capability of CNO nanoflakes facilitates the realization of complex logic devices with pristine interfaces, including NOT (inverter) and NAND gates. Figure 5a displays the optical image of the inverter device structure consisting of two series‐connected top‐gated MoS_2_ FETs with channel widths of 3 µm. The interconnection between the gate and source electrodes of the upper transistor establishes a pull‐up resistor configuration within the circuit. When a negative input voltage (*V*_IN_, representing logic ‘0’) is applied, the bottom transistor enters the OFF‐state, generating a high output voltage (*V*_OUT_), corresponding to logic ‘1’. Conversely, when a positive *V*_IN_ is applied (logic ‘1’), the bottom transistor is activated, driving *V*_OUT_ to ground potential (logic ‘0’), thereby successfully implementing NMOS inverter functionality [68]. The voltage transfer characteristics presented in Figure 5b exhibit sharp switching behavior across the full range of applied supply voltages (*V*_DD_ = 0.2‐1.0 V), with the notable absence of hysteresis during bidirectional voltage sweeps. The voltage gain, derived from the slope (‐d*V*_OUT_/d*V*_IN_) and serving as a critical performance parameter, achieves a value of ∼20 at *V*_DD_ = 1.0 V (Figure 5c). The value significantly surpasses unity, and a total noise margin of up to 89% is achieved at *V*_DD_ = 1.0 V (Figure S36a), indicating its robustness against noise and suitability for implementation in cascaded logic architectures. Furthermore, the device demonstrates exceptional power efficiency with peak static power consumption measurements remaining below 0.02 µW (Figure S36b,c), highlighting the advantages of CNO dielectrics for low‐power logic applications. Building upon the inverter design, we successfully fabricated a more complex NAND logic gate through integration of multiple top‐gated MoS_2_ FETs. The schematic diagram, circuit diagram, and the optical image are shown in Figure 5d,e. In this configuration, input signals *V*_IN1_ and *V*_IN2_ control the conduction states of respective transistors, while *V*_OUT_ monitors the resulting voltage drops. When either input *V*_IN1_ or *V*_IN2_ is at logic ‘0’, either one or both bottom transistors are in the OFF‐state, producing a high‐level *V*_OUT_ corresponding to logic ‘1’. In contrast, only when both inputs simultaneously reach logic ‘1’ does the output voltage drop to a low level, resulting in logic ‘0’. The logic operations and output results are shown in Figure 5f at a low *V*_DD_ of 1.0 V. The (0, 0), (1, 0), and (0, 1) inputs result in a stable output of 1 V with minimal fluctuation. In addition, dynamic switching characteristics in Figure 5g reveal excellent cycle stability across four sequential logic combinations. To evaluate the reproducibility of the logic devices, we fabricated an additional inverter and a NAND gate, both of which also demonstrated favorable performance (Figures S37 and S38). The successful realization of both NOT and NAND logic gates conclusively validates the suitability of CNO dielectrics for integrated logic circuit applications.

## 3 Conclusion

In summary, we demonstrate that perovskite‐like ternary niobium oxides CNO, KNO, and NNO exhibit excellent air stability, wide bandgaps, high dielectric constants, and high breakdown field strengths, placing them in a comparable position among the state‐of‐the‐art dielectrics. We elucidate that these properties are determined by a general Nb‐O orbital hybridization mechanism with highly ionic intercalated ions. Utilizing these materials as gate dielectrics, MoS_2_ FETs achieve exceptional performance metrics: high on/off current ratios (exceeding 10^7^), ultralow bottom‐gate leakage currents (lower than 10^−6^ A cm^−2^), and SS approaching the theoretical limit (∼60 mV dec^−1^), concurrently attributed to their high crystalline quality and well‐defined vdW heterointerfaces. We have further validated their technological potential through the demonstration of high‐performance inverters and NAND logic gates employing CNO dielectrics. The remarkable dielectric properties and device performance of these materials provide theoretical support for the development and application of high‐performance, low‐power 2D dielectrics. Moreover, these materials can be further optimized toward ultrathin, wafer‐scale growth, making them a promising platform for scalable 2D FETs in integrated circuits.

## 4 Methods

### 4.1 Synthesis of 2D CNO, KNO, and NNO Nanoflakes

2D CNO nanoflakes were synthesized via a substrate‐assisted molten salt strategy. A homogeneous mixture of CaCl_2_·2H_2_O (80 mg, 99.99%, Aladdin) and Nb_2_O_5_ (50 mg, 99.99%, Aladdin) was mechanically milled with 200 mg of LiCl‐KCl eutectic salt (41.8 mol% KCl, 99.99%, Aladdin). The resulting powder was dispersed onto freshly cleaved mica substrates (Changchun City Taiyuan Fluorphlogopite Co., Ltd.) and loaded into an alumina boat. The reaction was carried out in a 1‐inch diameter quartz tube positioned within a single‐zone furnace (SK‐B02123K‐200, Zhonghuan Electric Furnace Co., Ltd.). The temperature was elevated to 660°C at a ramp rate of 10°C/min under an argon flow (50 sccm) and maintained for 10 min. After cooling to ambient temperature, the mica substrates were rinsed repeatedly with deionized water to eliminate residual salts and impurities. When the precursor was placed under the mica, it will cause vapor phase growth at a higher temperature of 750°C. For the synthesis of 2D KNO nanoflakes, a mixture of KCl (140 mg) and Nb_2_O_5_ (30 mg) was homogenized by mechanical milling and evenly distributed in an alumina boat, with mica substrates placed atop the powder for growth. The furnace temperature was increased to 850°C (10°C/min) under an argon atmosphere (50 sccm) and held for 10 min. For the KNO crystals synthesized using a substrate‐assisted molten salt strategy, part of the mixture of KCl (90 mg) and Nb_2_O_5_ (30 mg) was sprinkled on mica, and the reaction temperature was 800°C. For NNO nanoflakes, a NaCl‐KCl eutectic mixture (180 mg, 50.6 mol% NaCl, 99.99%, Aladdin) was employed as both the sodium source and a flux to reduce the growth temperature, in combination with 30 mg of Nb_2_O_5_. The precursor mixture was uniformly milled and dispersed in a corundum boat, and mica substrates were positioned above the powder. Growth was conducted at 850°C for 10 min under identical argon flow conditions. For the NNO crystals grown using the substrate‐assisted molten salt strategy, a portion of the mixture was sprinkled onto mica, and the reaction was conducted at 770°C, while all other conditions remained the same.

### 4.2 Transfer of 2D Nanoflakes

The CNO, KNO, and NNO nanoflakes were transferred to arbitrary substrates via dry transfer processes. Due to their weak adhesion to the mica substrate, CNO and KNO nanoflakes can be directly transferred using PDMS. A PDMS stamp was placed onto the mica surface to adhere the nanoflakes and then peeled off. The nanoflakes can be relocated onto the PDMS. The stamp was then aligned with the target substrate using a precision transfer stage (Metatest, E1‐T). To facilitate release, the stack was heated at 60°C for 1 min to soften the PDMS, which was subsequently lifted, leaving the nanoflakes on the target substrate. For NNO nanoflakes, which bond more strongly to mica, a polyvinyl butyral (PVB)‐assisted transfer method was employed. A thin PVB film was prepared by spin‐coating a 2 wt.% PVB solution onto a glass disc and drying it at 80°C. A small section of the PVB film was detached and laminated onto a PDMS stamp, which was then aligned and pressed onto the nanoflakes on mica. The assembly was heated at 65°C for 2 min to enhance adhesion between the PVB and the nanoflakes. Upon peeling the PDMS away, the PVB film remained on the mica. After cooling, the film was carefully delaminated, carrying the nanoflakes, and transferred to the target substrate using the same alignment and heating process. Finally, the PVB was dissolved in ethanol at 45°C, leaving the NNO nanoflakes on the substrate.

### 4.3 Characterizations

The structural and chemical composition of the synthesized nanoflakes was comprehensively characterized using a suite of analytical techniques. Morphological and thickness analyses were conducted via optical microscopy (Nexcope NM910) and AFM (Cypher S, Oxford Instruments Asylum Research), respectively. XPS (Kratos Analytical AXIS‐Ultra) was employed to determine the chemical composition of the CNO. Raman spectroscopy measurements were performed using a confocal microscope spectrometer (WITEC alpha300 R) equipped with a 532 nm excitation laser and a 300 grooves/mm grating. For nanoscale structural and compositional analysis, HRTEM and EDS were carried out on a FEI Tecnai F20 microscope. HAADF‐STEM was performed using a spherical‐aberration‐corrected microscope (Nion U‐HERMES200). Cross‐sectional specimens of CNO nanoflakes were prepared via FIB milling (Scios FEI). Optical absorption properties were evaluated using a micro‐area UV–vis confocal spectrometer (MStarter ABS Metatest) in transmission mode.

### 4.4 Dielectric Characterizations

The MIM devices were fabricated using two types of bottom electrodes: metallic and graphite‐based. For the metallic electrodes, patterned Cr/Au (10/20 nm) structures with varying widths were deposited on quartz substrates through a combination of UV photolithography (Suss MA6 MicroTec AG) and electron‐beam evaporation (TEMD500, Beijing Technol Science Co., Ltd). In parallel, mechanically exfoliated graphite electrodes were prepared on sapphire substrates. The dielectric layers, CNO, KNO, and NNO nanoflakes, were precisely aligned and transferred onto the bottom electrodes using the dry transfer technique mentioned before. For the top electrode fabrication, Au (100 nm) was deposited onto pre‐patterned Si substrates through photolithographic processing. These top electrodes were subsequently transferred onto the nanoflakes using the PVB‐assisted method. The current density versus voltage and capacitance versus voltage characteristics were systematically measured at room temperature using a semiconductor parameter analyzer (Keysight 4200A) integrated with a probe station (Lake Shore CRX‐6.5K).

### 4.5 Device Fabrication and Characterizations

For the top‐gate FET, few‐layer MoS_2_ was mechanically exfoliated onto marked Si/SiO_2_ substrates, followed by the precise dry transfer of the CNO dielectric layer. The drain, source, and gate electrodes (Cr/Au 5/45 nm) were subsequently fabricated in via electron‐beam lithography (EBL) and electron‐beam evaporation. For the bottom‐gate FET, patterned Cr/Au (10/20 nm) bottom‐gate electrodes were first deposited on Si/SiO_2_ substrates using UV photolithography and electron‐beam evaporation. The dielectric layer and a few‐layer MoS_2_ were sequentially transferred onto the electrode. Finally, pre‐patterned Au source/drain electrodes (100 nm) were aligned and transferred via a PVB‐assisted method. For logic applications, Cr/Au (5/15 nm) electrodes were patterned on exfoliated MoS_2_ using EBL and evaporation. After transferring the CNO dielectric layer, Cr/Au (5/45 nm) top electrodes were deposited via the same EBL and evaporation process. All the measurements were systematically conducted at room temperature using a semiconductor parameter analyzer (Keysight 1500A) integrated with a probe station (Lake Shore CRX‐6.5K).

### 4.6 Theoretical Calculations

All DFT calculations were performed using the Vienna ab initio Simulation Package (VASP version 5.4.1) [69]. Electron‐core interactions were modeled with projector augmented wave (PAW) [70] pseudopotentials, while valence electrons were treated using a plane‐wave basis set. Exchange‐correlation effects were approximated with the Perdew‐Burke‐Ernzerhof (PBE) [71] functional within the generalized gradient approximation (GGA) framework. For surface energy calculations, the Monkhorst‐Pack *k* mesh of 6 × 6 × 1 was adopted to represent the reciprocal space of the structures. The surface slabs were stabilized through adsorption of oxygen atoms to simulate ambient conditions, and the energy was defined as *E*_surf_ = (*E*_2L_ ‐ 2*E*_unit_ + *mµ*_O_)/2*A*, where *E*_unit_ and *E*_2L_ denote bulk and slab energies, respectively, *A* represents surface area, *m* was the number of additional oxygen atoms on the surface, and *µ*_O_ was the chemical potential of oxygen. For the calculations of electronic structure, the Heyd‐Scuseria‐Ernzerhof (HSE06) [72] methods are used, with *k*‐point meshes of 7×17×19 for CNO, 18×3×9 for KNO, and 21×21×8 for NNO. The band alignments were calculated by extracting the electrostatic potential of the vacuum region in the slab models, for which the vacuum layers were set to a thickness of 20 Å. All computations employed a 520‐eV plane‐wave cutoff energy with convergence thresholds of 10^−5^ eV for total energy and 0.01 eV/Å for ionic forces.

## Author Contributions

Y.C. conceived and supervised the project. B.Z. designed the experiments, synthesized the samples, performed material characterization, fabricated the devices, conducted electrical measurements, and carried out theoretical calculations. J.G. designed the logic devices and performed the corresponding electrical measurements. J.Y., J.L., C.W., and Z.X. assisted with material characterization and device fabrication. J.W., G.Z., and Y.H. assisted with the theoretical aspects and analyzed the experimental data. B.Z., J.G., and Y.C. co‐wrote the manuscript. All authors discussed the results and commented on the manuscript.

## Conflicts of Interest

The authors declare no conflicts of interests.

## Supporting information




**Supporting File**: adma72107‐sup‐0001‐SuppMat.docx

## Data Availability

The data that support the findings of this study are available from the corresponding author upon reasonable request.
