# Supplementary material for: High‐κ Perovskite‐Like Ternary Niobium Oxide Dielectrics for 2D Electronics
Source: Adv Mater. 2026 Jan 8;38(11):e20423. doi: 10.1002/adma.202520423 (PMC12921349; doi:10.1002/adma.202520423)
Supplement: Supplementary file 1 — Supporting File: adma72107‐sup‐0001‐SuppMat.docx [file ADMA-38-e20423-s001.docx]

Supporting Information

**High-κ Perovskite-like Ternary Niobium Oxides for Two-dimensional Electronics**

*Biao Zhang^1,2,#^, Jianmiao Guo,^1,2,#^ Jianmin Yan^1,2^,* *Jialiang Wang^1,2^, Chao Yun^3^, Guang Zeng^1,2^, Jie Li^1,2^, Cong Wang^1,2^, Zhengdao Xie^1,2^, Yanglong Hou^3^, and Yang Chai^1,2,^**

^1^ Department of Applied Physics, The Hong Kong Polytechnic University, Hong Kong, China

^2^ Joint Research Center of Microelectronics, The Hong Kong Polytechnic University, Hong Kong, China

^3^ School of Materials, Shenzhen Campus of Sun Yat-Sen University, Shenzhen, China

# These authors contribute equally

*Corresponding author. Email: ychai@polyu.edu.hk

**Supplementary Note I. Crystal structures of the ternary niobium oxides**

Ternary niobium oxides have various element ratios and different structures. CaNb_2_O_6_ (CNO) crystallizes in an orthorhombic columbite-type structure with Pbcn space group (**Figure S1a, d**). NbO_6_ octahedra form edge-sharing networks along the *b*-*c* planes, creating negatively charged layers. These layers are separated by Ca^2+^ cations along the *a*-axis direction.^[1]^ KNb_3_O_8_ (KNO) crystal possesses an orthorhombic structure (space group Cmcm) composed of similar NbO_6_ octahedral sheets along the *a*-*c* planes, with K^+^ cations occupying the interlayer spaces (**Figure S1b, e**).^[2]^ CNO and NNO exhibit distinct crystallographic arrangements of their NbO_6_ octahedra and charge-balancing cations, resulting in different space group symmetries. In particular, the Na_2_Nb_4_O_11_ (NNO) crystal investigated in this work adopts a previously unreported hexagonal structure (space group P6_3_22), as determined through first-principles calculations. This novel framework consists of alternating layers containing two coordination geometries, including the edge-sharing pentagonal NbO_7_ bipyramids and interconnected NaO_7_ polyhedra and NbO_6_ octahedra (**Figure S1c, f**).

**
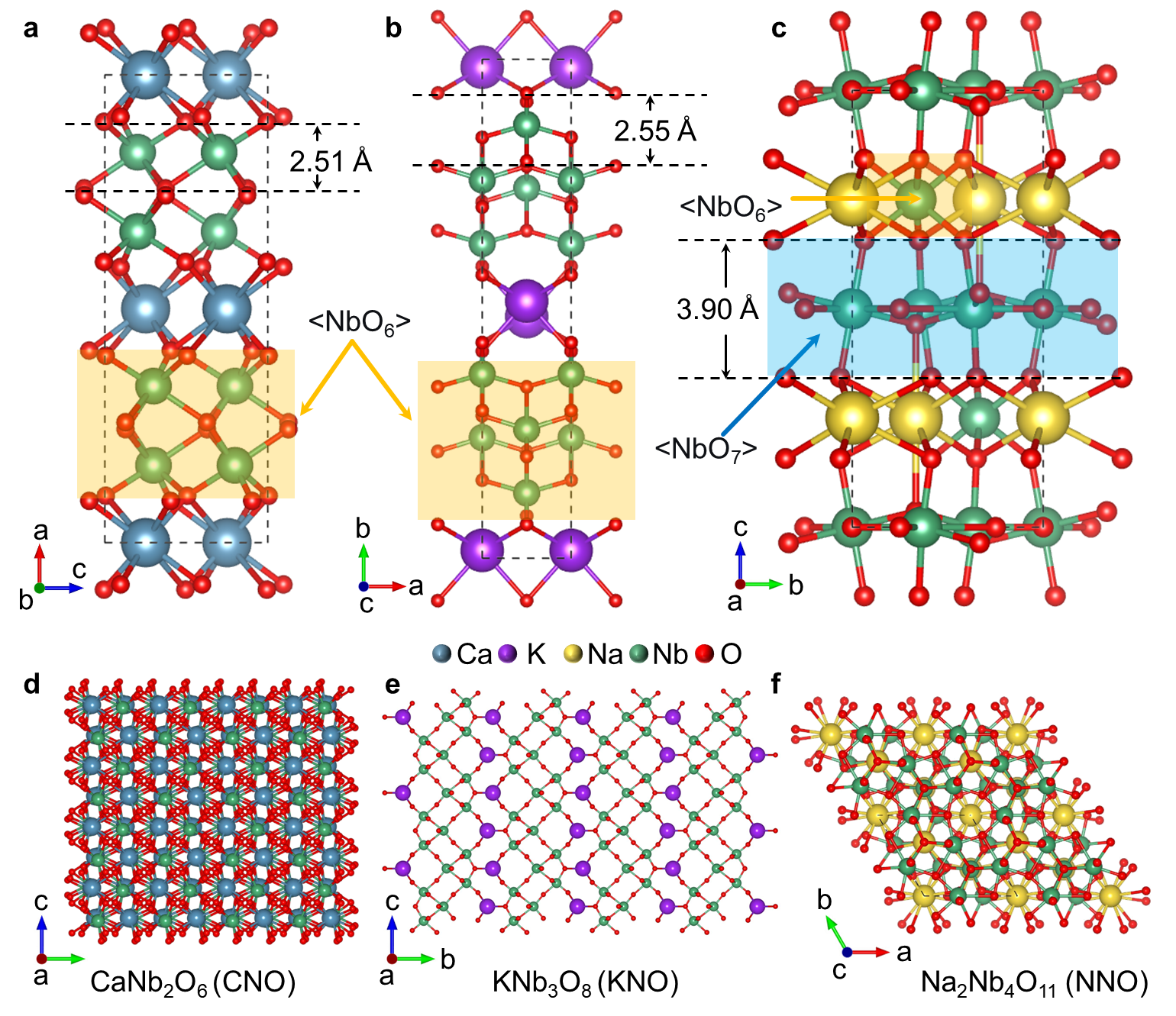
**

**Figure S1.** Crystal structure of the ternary niobium oxides. (a,d) CNO. (b,e) KNO. (c,f) NNO.


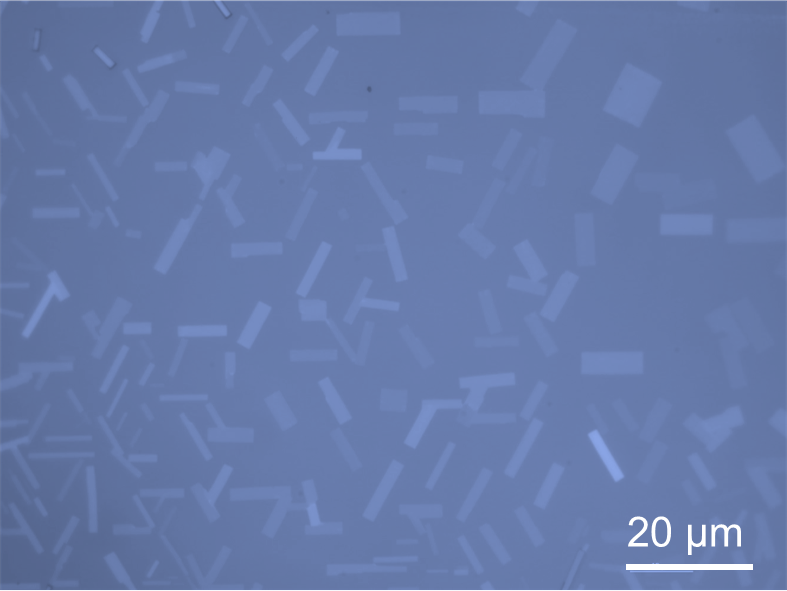


**Figure S2.** Small crystalline CNO domains synthesized by CVD process.


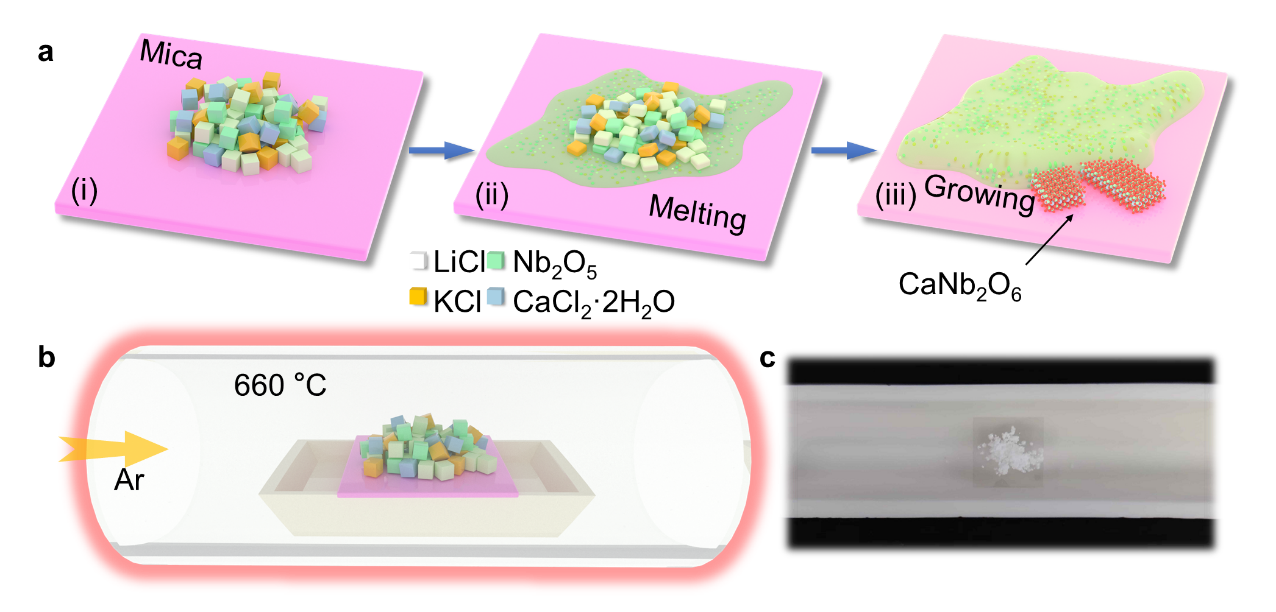


**Figure S3.** Synthesis of CNO nanoflakes via molten eutectic salt method. (a) Schematic illustration of the growth process and mechanism. (b) Experimental configuration for the synthesis of 2D CNO crystals. (c) Optical image of precursor materials and growth substrate for CNO synthesis.


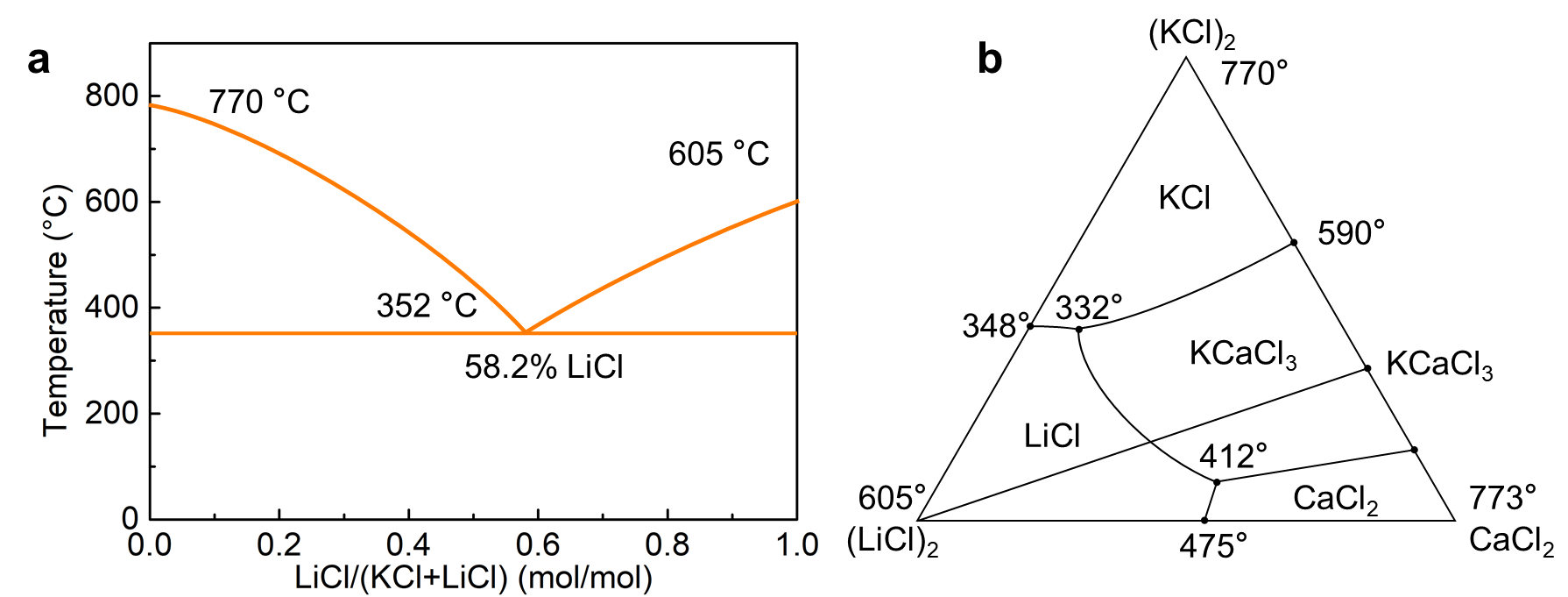


**Figure S4.** Phase diagrams of eutectic salts. (a) LiCl-KCl eutectic system. (b) LiCl-KCl-CaCl_2_ eutectic system.


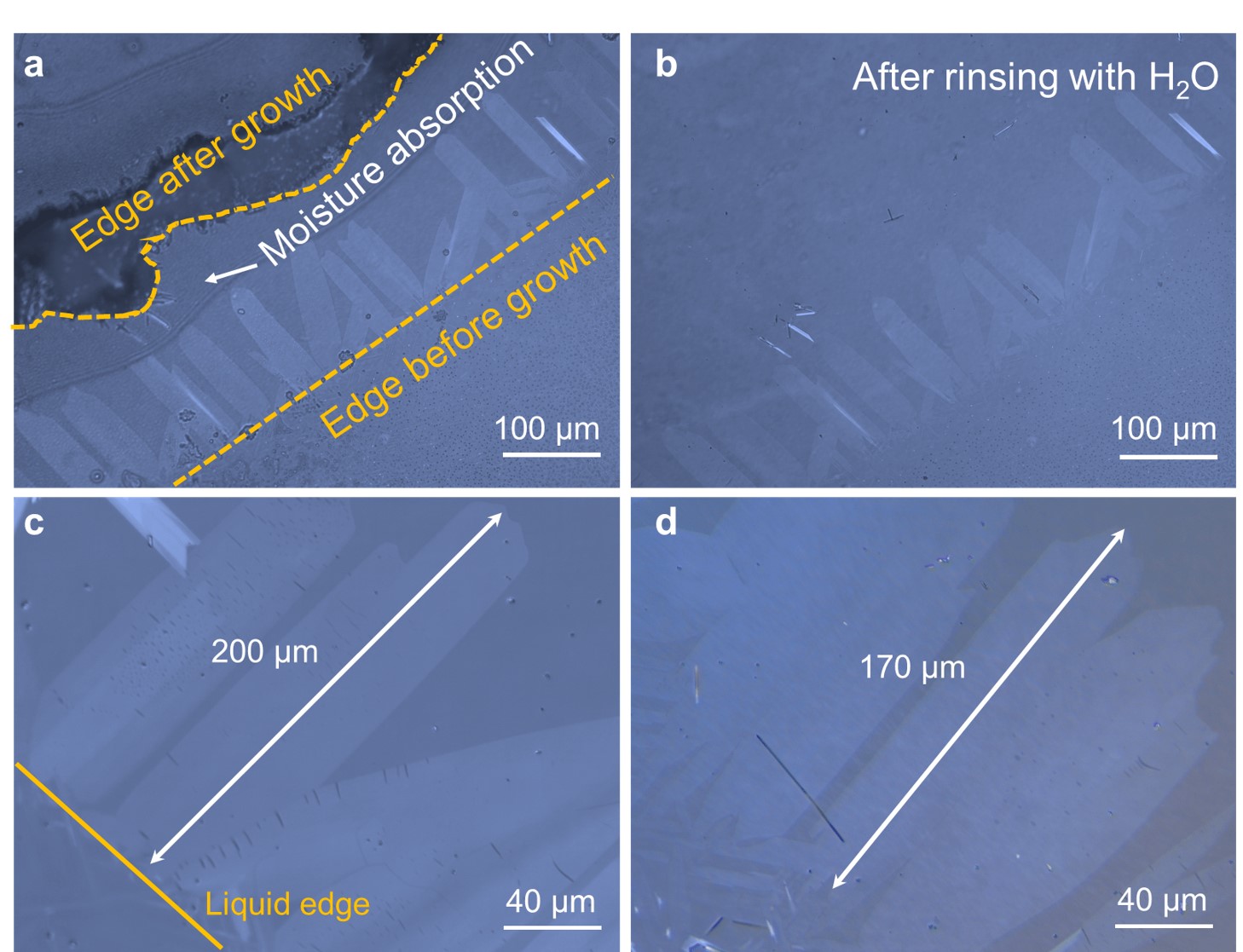


**Figure S5.** Synthesis and morphology of CNO nanoflakes. (a) Optical image of CNO nanoflakes synthesized via substrate-assisted molten salt growth. (b) Corresponding optical image after salt removal. (c,d) Large-area CNO nanoflakes.


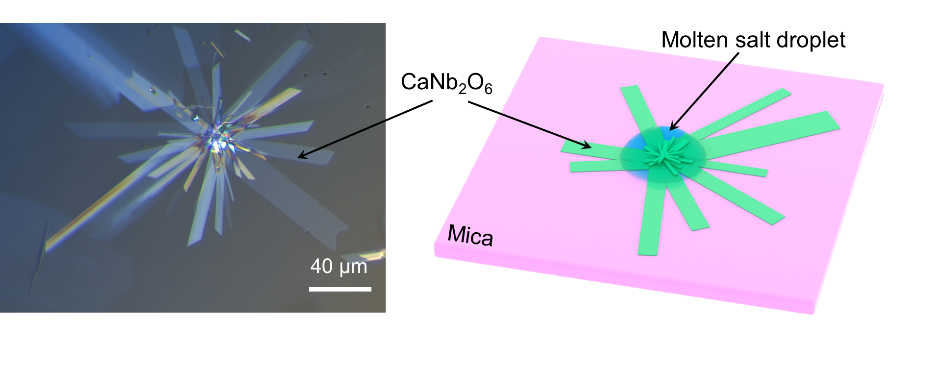


**Figure S6.** CNO nanoflakes grow via central nucleation with random orientations, along with a corresponding schematic diagram of the growth mechanism.


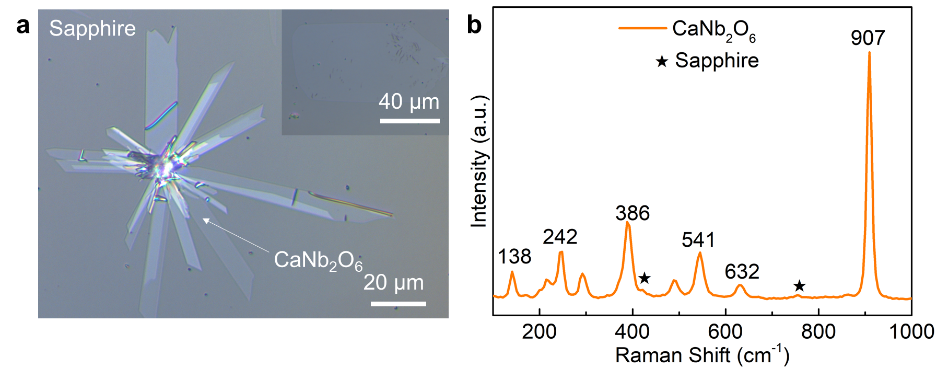


**Figure S7.** The CNO nanoflakes synthesized on sapphire substrate. (a) Optical image of the CNO nanoflakes. (b) Raman spectrum of CNO grown on sapphire.


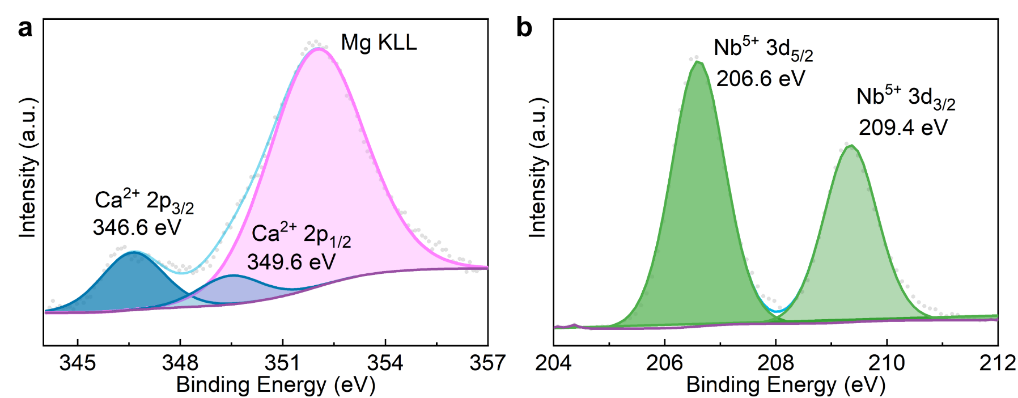


**Figure S8.** XPS characterization of the CNO nanoflakes. High-resolution XPS patterns of (a) Ca 2*p* and (b) Nb 3*d* orbitals. The Ca 2*p* spectrum exhibits characteristic spin-orbit doublet components at 346.6 eV (Ca 2*p*_3/2_) and 349.6 eV (Ca 2*p*_1/2_), while the Nb 3*d* spectrum shows corresponding doublet peaks at 206.6 eV (Nb 3*d*_5/2_) and 209.4 eV (Nb 3*d*_3/2_).^[3]^ The XPS peak of Mg originates from mica.


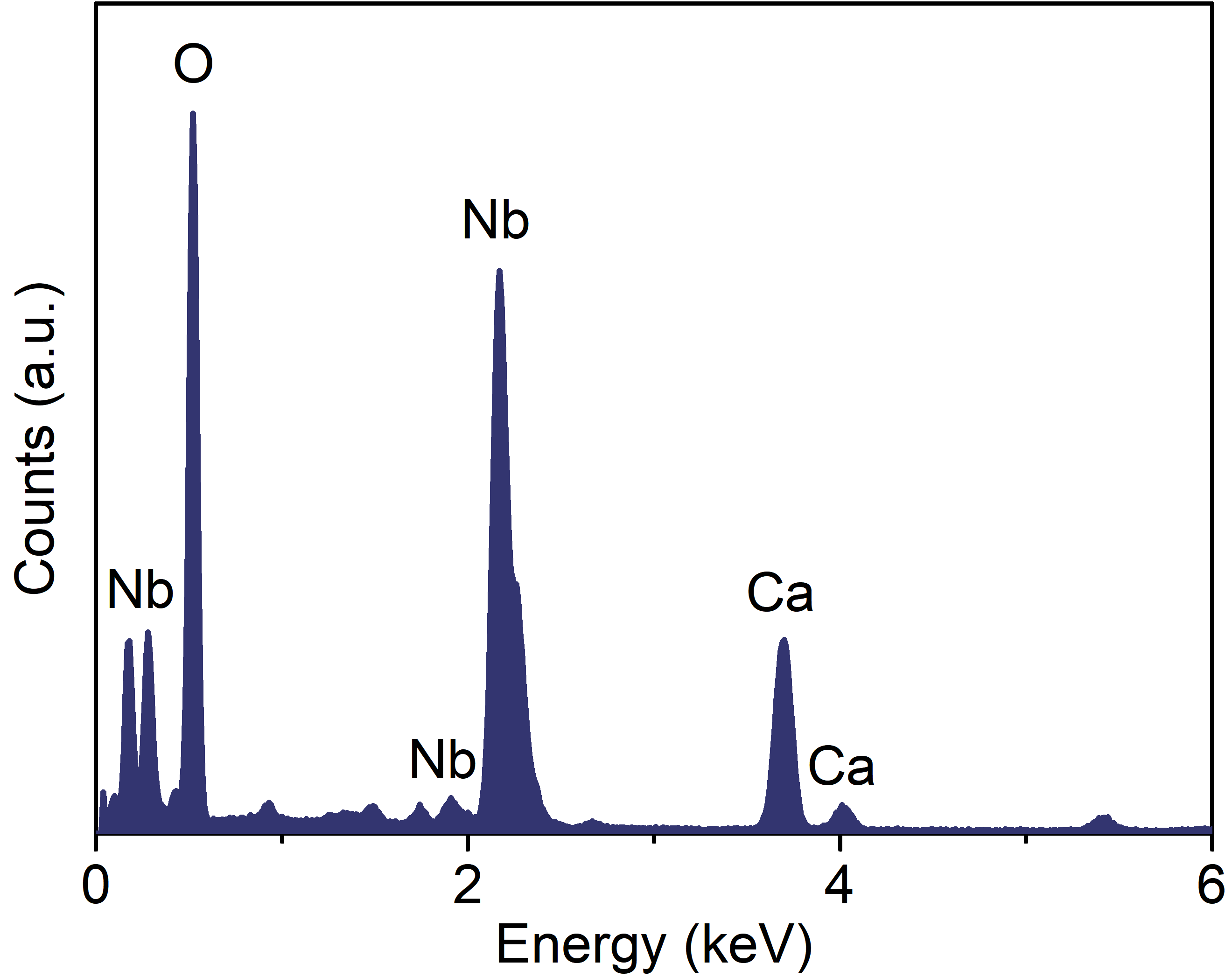


**Figure S9.** Representative EDS spectrum of CNO nanoflakes.


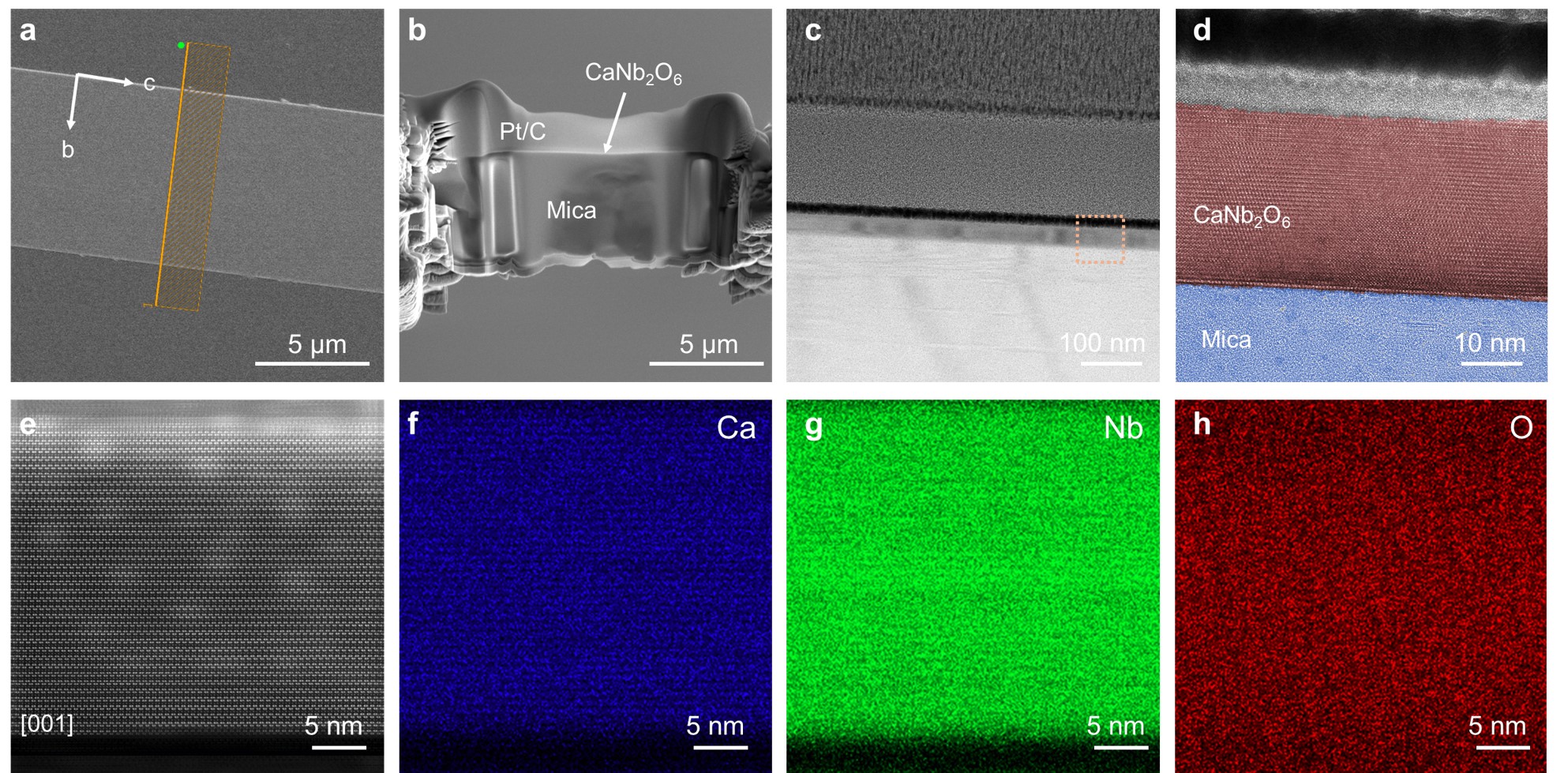


**Figure S10.** (a) Scanning electron microscopy image of the sample prepared for FIB milling, with the milling direction aligned along the short side (*b*-axis of the crystal). (b) Low-magnification TEM image of the FIB-prepared cross-sectional sample. (c,d) Cross-sectional TEM image of CNO nanoflake on mica. (e-h) HAADF-STEM cross-sectional image (e) and corresponding EDS elemental mappings (f-h).


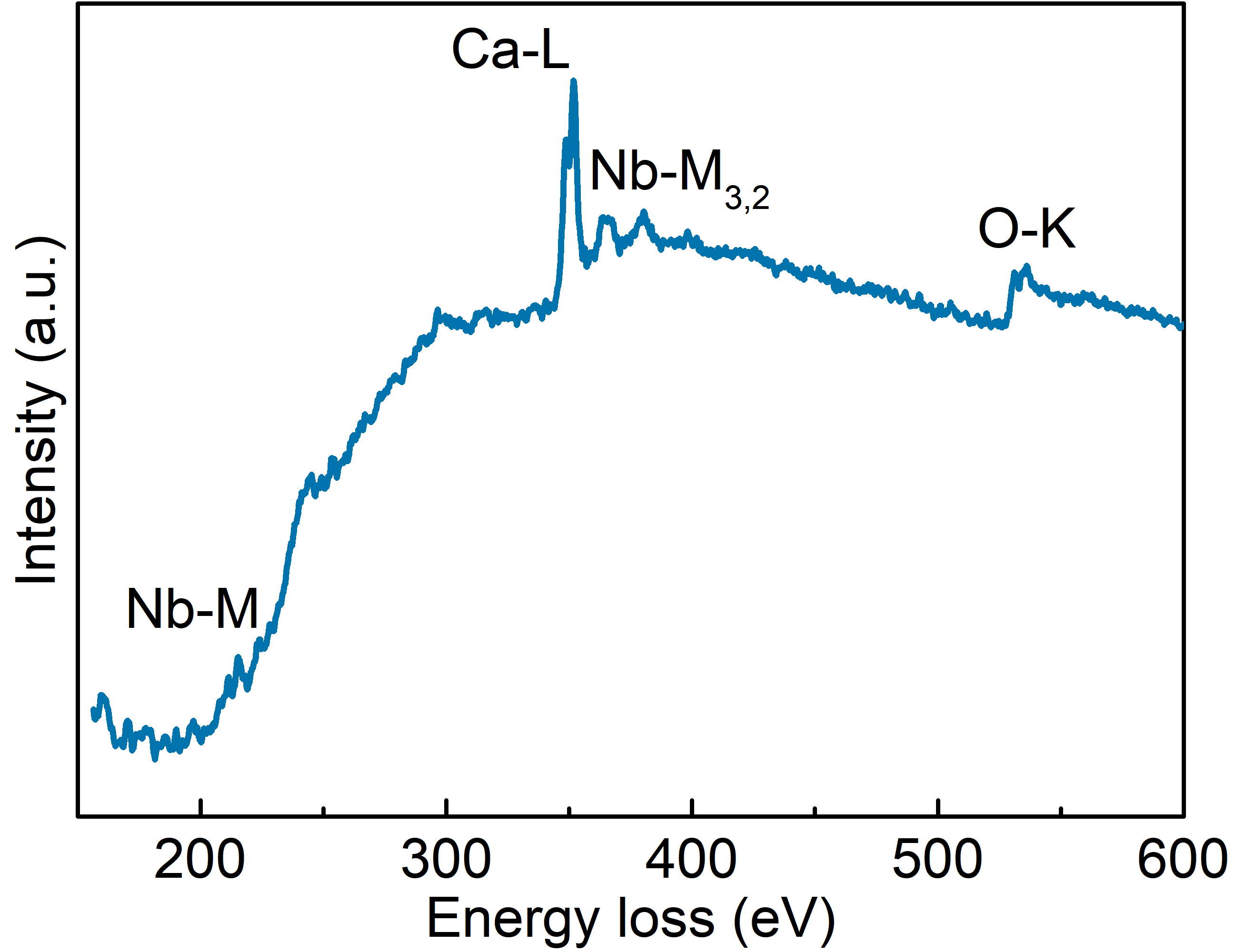


**Figure S11.** EELS spectrum of O K-edge, Nb M-edge, Nb M_3,2_-edge and Ca L-edge core-level excitations.


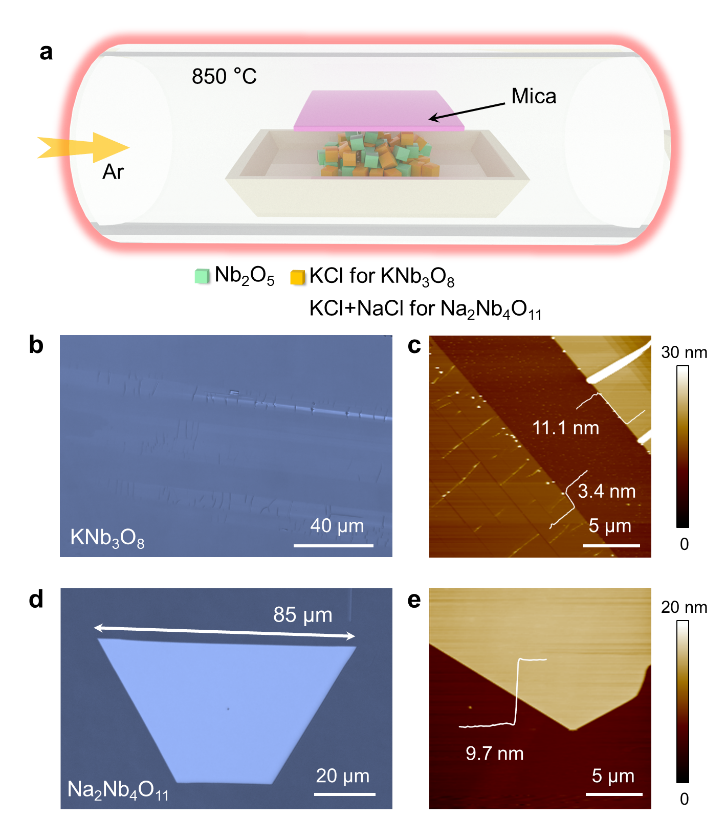


**Figure S12.** Synthesis and characterization of KNO and NNO nanoflakes. (a) Schematic of the experimental setup for 2D KNO and NNO crystal growth. (b,d) Optical images of as-synthesized KNO (b) and NNO (d) nanoflakes. (c,e) AFM topography images with corresponding height profiles of ultrathin KNO (c) and NNO (e) nanoflakes.


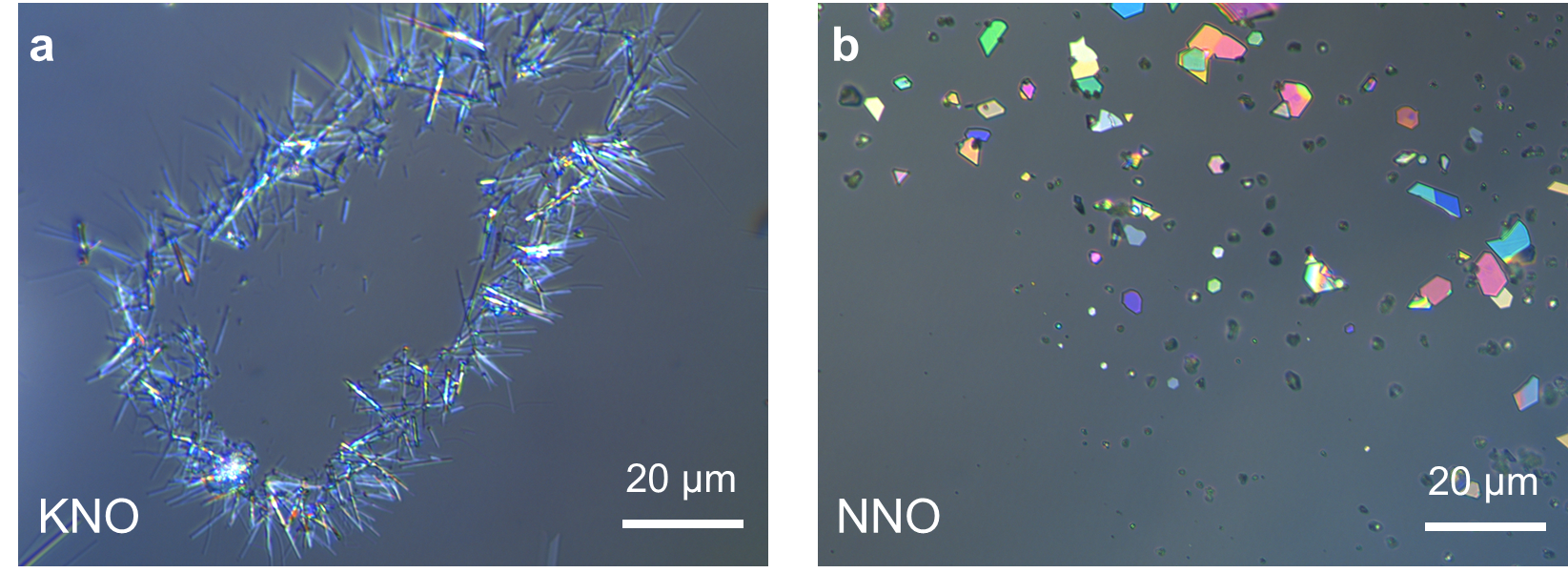


**Figure S13.** KNO and NNO crystals grown via the substrate-assisted molten-salt eutectic growth strategy. (a) Stacked KNO nanowires exhibiting limited dimensions. (b) NNO nanoflakes with non-uniform thickness and size.


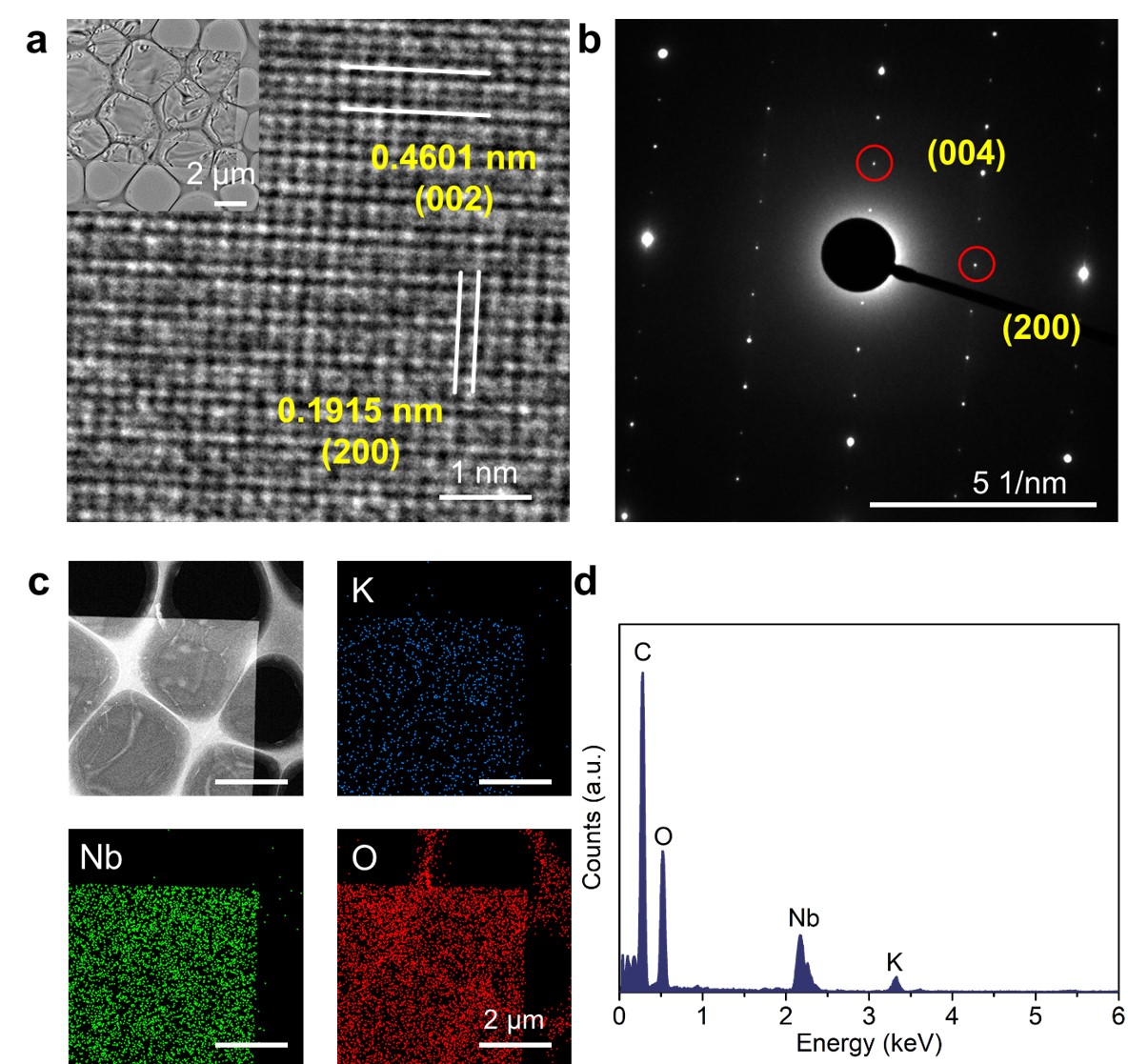


**Figure S14.** Structural and chemical composition characterization of KNO nanoflakes. (a) HRTEM image of the KNO nanoflake. Inset: Low-magnification TEM overview. (b) Corresponding SAED pattern. (c) Low-magnification ADF-STEM image with EDS element mappings of K, Nb, and O. (d) Corresponding EDS spectrum.


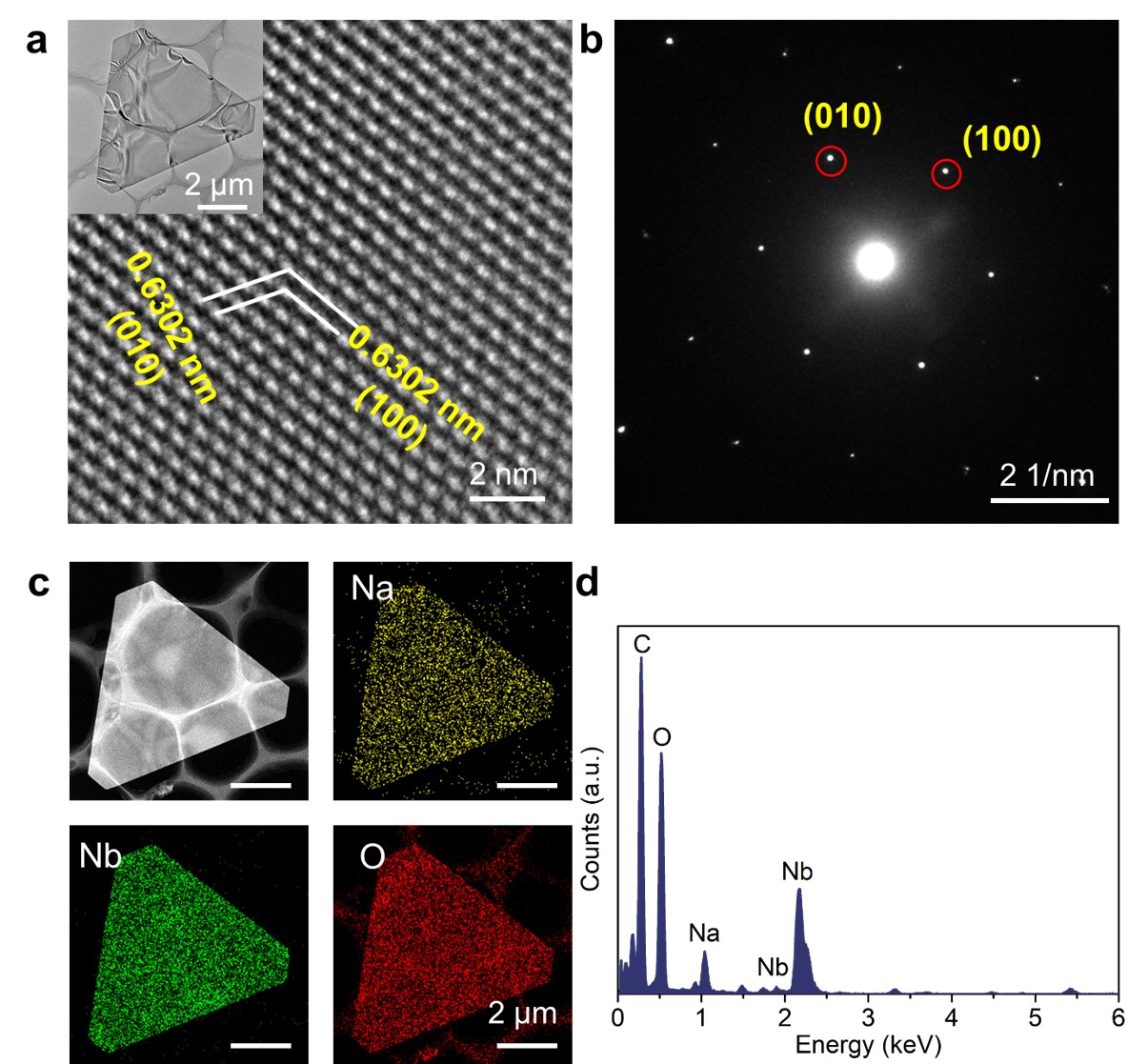


**Figure S15.** Structural and chemical composition characterization of NNO nanoflakes. (a) HRTEM image of the NNO crystal. Inset: Low-magnification TEM image. (b) Corresponding SAED pattern. (c) ADF-STEM image and EDS element mappings of Na, Nb, and O. (d) Corresponding EDS spectrum.

**Supplementary Note II. Atomic structure characterization of NNO crystal.**

Given the previously unreported crystal structure of NNO, we conducted comprehensive HAADF-STEM analysis to verify its atomic configuration (**Figure S16**). Large-area HAADF-STEM imaging shown in **Figure S16a, b** reveals a defect-free crystalline lattice with perfect long-range order. The atomic contrast in HAADF-STEM images follows *Z*-contrast principles, where the intensity is proportional to the square of the atomic number. Consequently, oxygen atoms with *Z* = 8 remain undetectable. Three distinct contrast levels corresponding to different atomic species are clearly resolved in **Figure S16c**. The atomic arrangement and crystal structure show perfect correspondence. The atomic arrangement perfectly matches the predicted crystal structure, with the darkest spots representing isolated sodium atoms, intermediate-intensity spots corresponding to single niobium atoms, and the brightest features indicating overlapping sodium-niobium atomic columns. In addition, atomic-resolution EDS mappings (**Figure S16d-g**) confirm sodium presence in both the brightest and darkest regions, as highlighted in the dashed box area. However, the dense packing of niobium atoms within the structure makes it challenging to resolve individual Nb atomic positions.


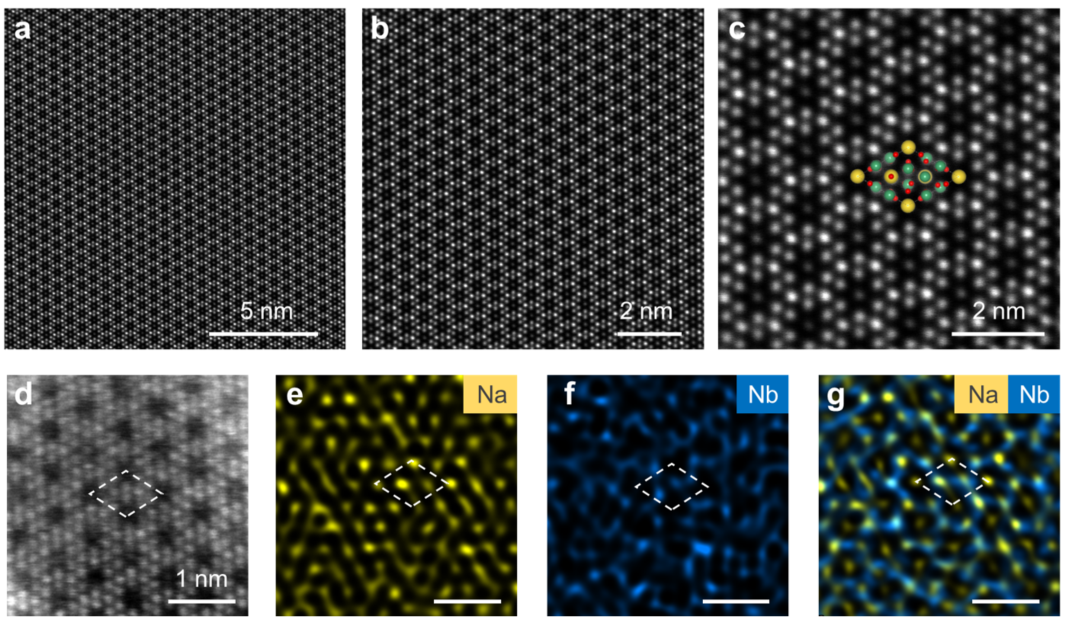


**Figure S16.** Atomic structure characterization of NNO crystal. (a,b) In-plane atomic-resolution HAADF-STEM images. (c) High-magnification HAADF-STEM image of the nanoflake. (d-g) ADF-STEM image and atomic-resolution elemental mappings.


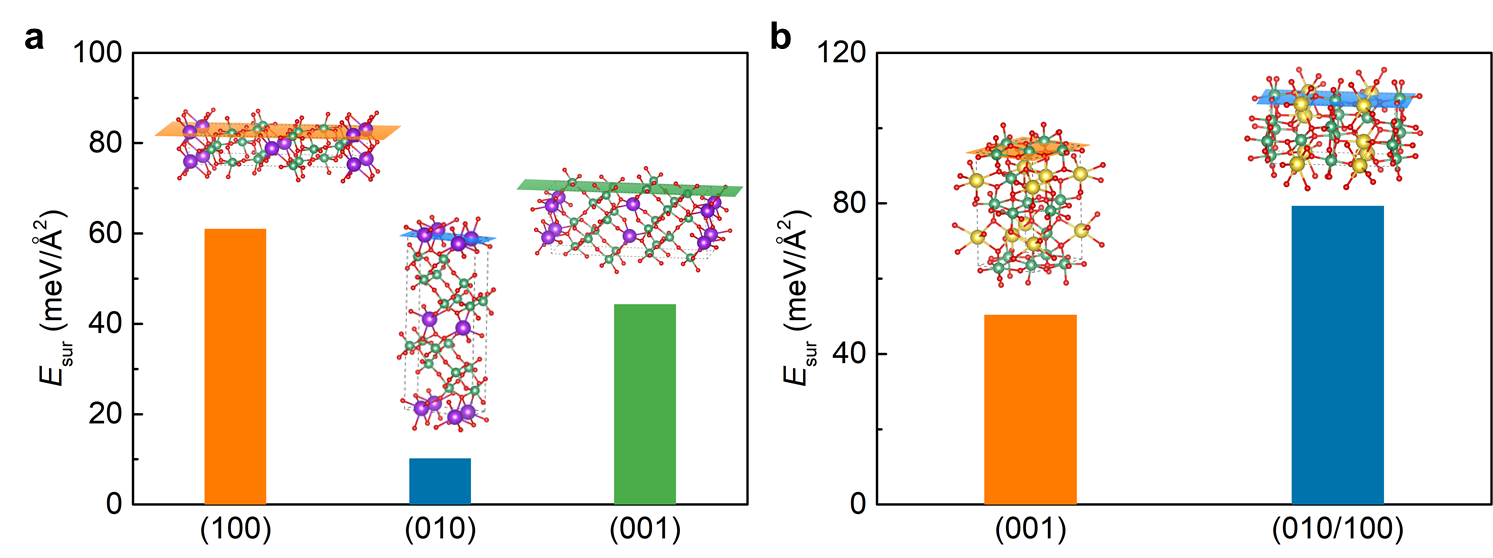


**Figure S17.** Calculated *E*_sur_ of KNO and NNO. (a) *E*_sur_ for the (100), (010), and (001) planes of KNO, with values of 61.03, 10.15, and 44.31 meV Å^-2^, respectively. (b) *E*_sur_ for the (001) and (010/100) planes of NNO, with values of 50.48 and 79.27 meV Å^-2^, respectively.


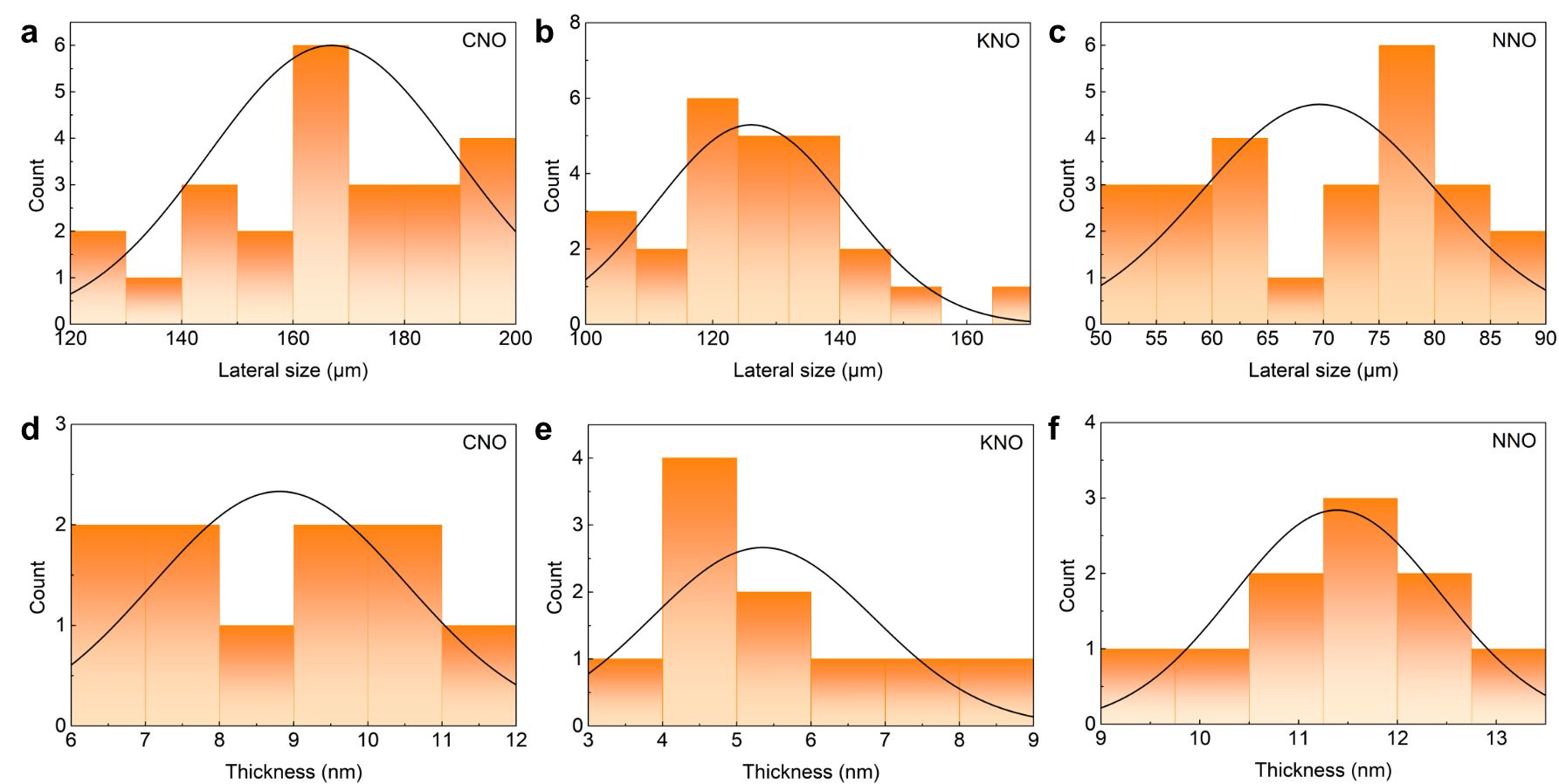


**Figure S18.** Size and thickness distributions of the nanoflakes. The histograms show the statistical distribution of (a-c) the maximum lateral size and (d-f) the minimum thickness, which correspond to (a, d) CNO, (b, e) KNO, and (c, f) NNO nanoflakes, respectively.


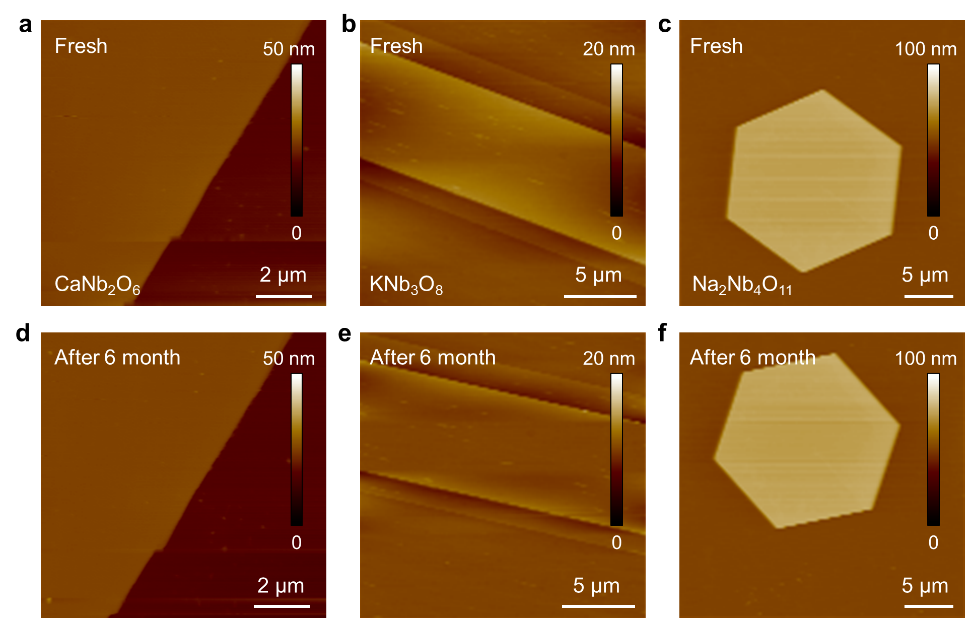


**Figure S19.** Atmospheric stability analysis of nanoflakes via AFM. (a-c) Topography images of as-synthesized nanoflakes. (d-f) Corresponding samples after 6 months of ambient air exposure.


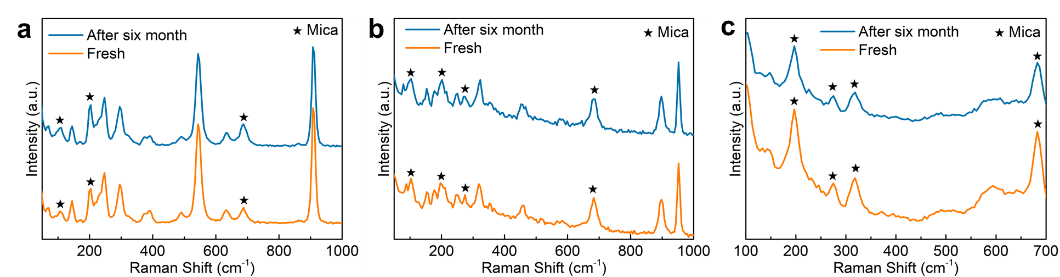


**Figure S20.** Raman spectroscopic analysis of air-stored nanoflakes after 6 months. (a) CNO, (b) KNO, and (c) NNO.


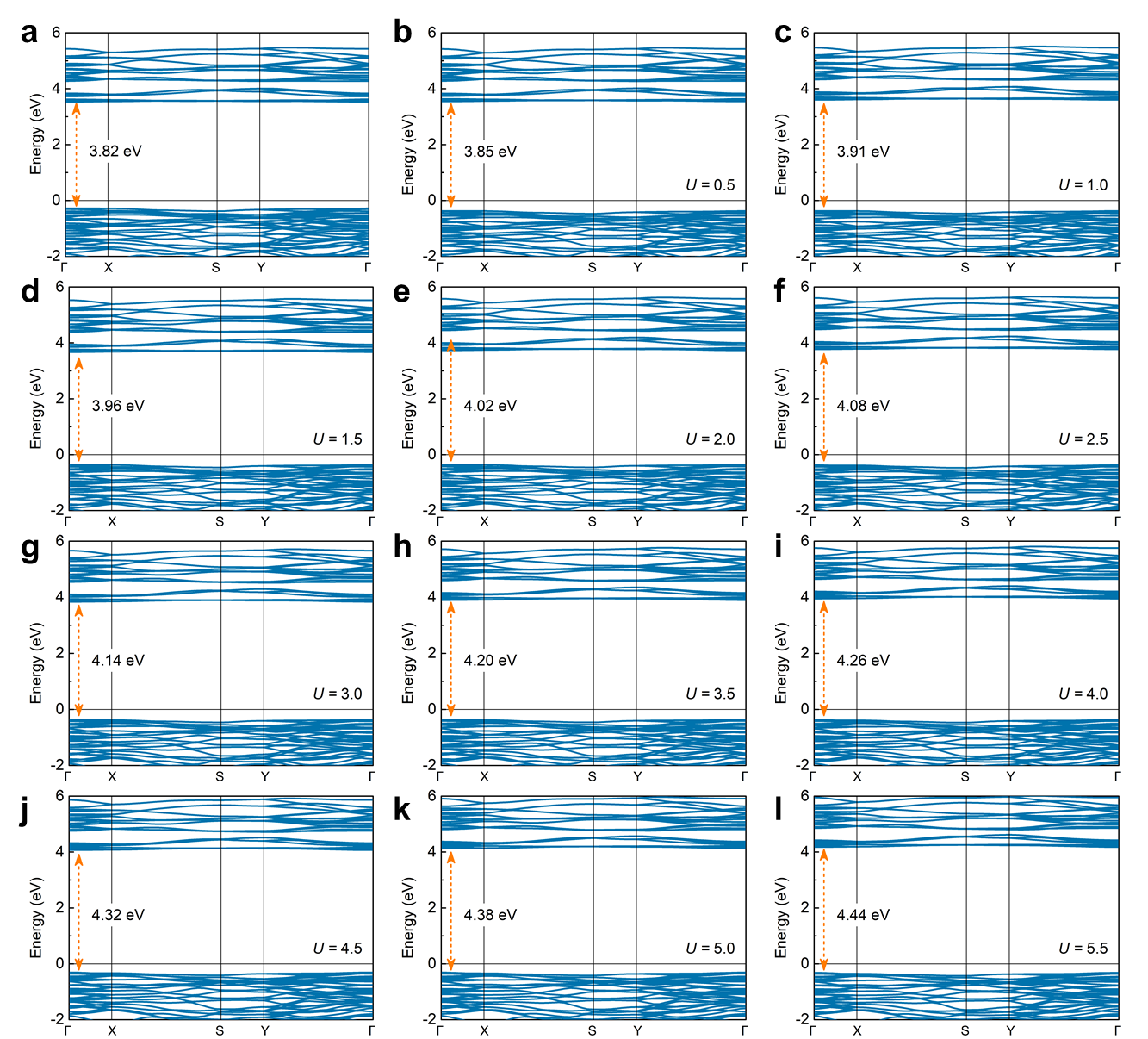


**Figure S21.** Electronic structure calculations of CNO crystals using standard GGA (a) and DFT + *U* method (b-l). The bandgap increases linearly from 3.85 eV at *U* = 0.5 eV to 4.44 eV at *U* = 5.5 eV.


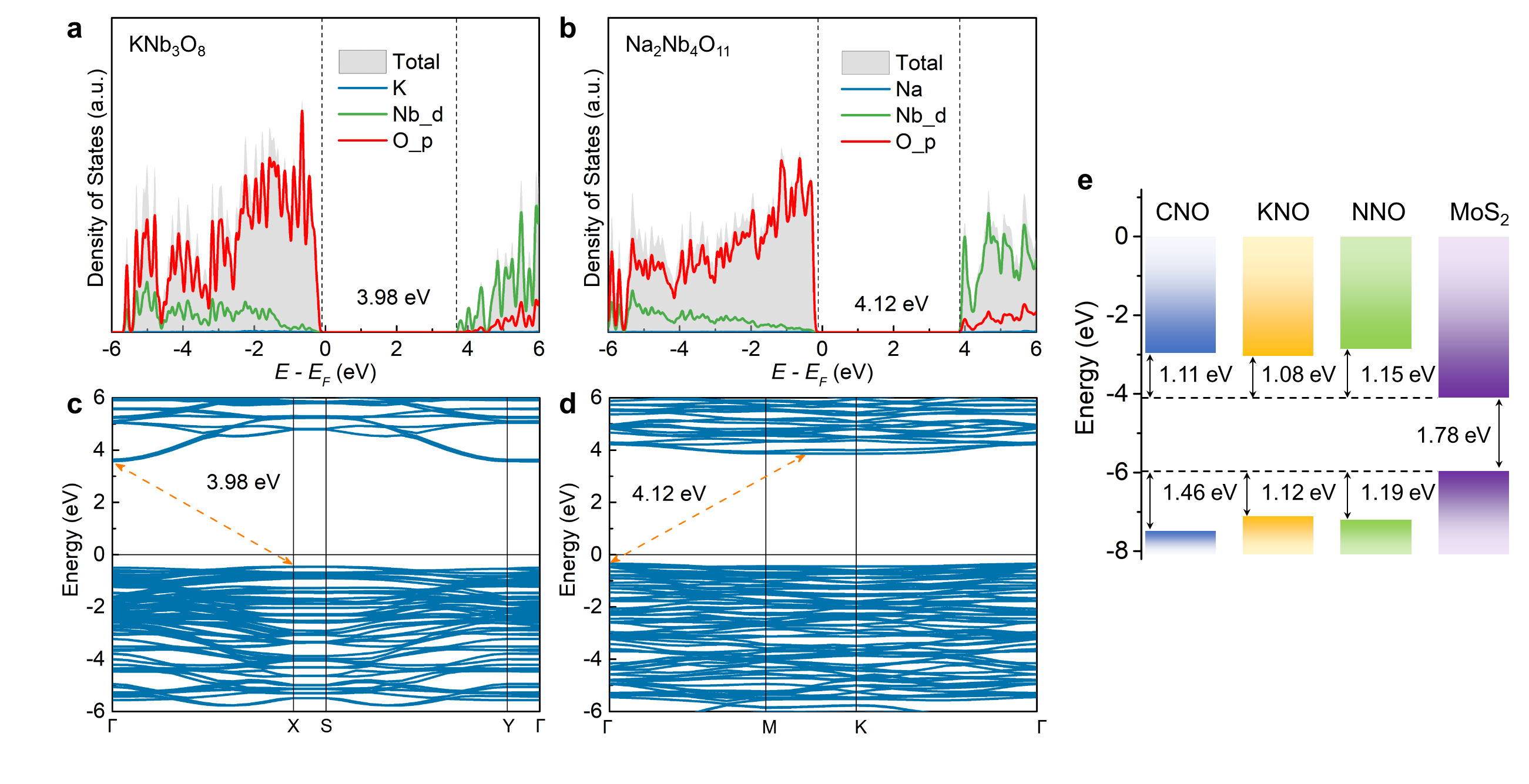


**Figure S22.** Electronic structure calculations of KNO and NNO crystals. (a,c) DOS (a) and band structure (c) of KNO. (b,d) DOS (b) and band structure (d) of NNO. (e) Calculated band alignments of CNO, KNO, and NNO relative to MoS_2_.


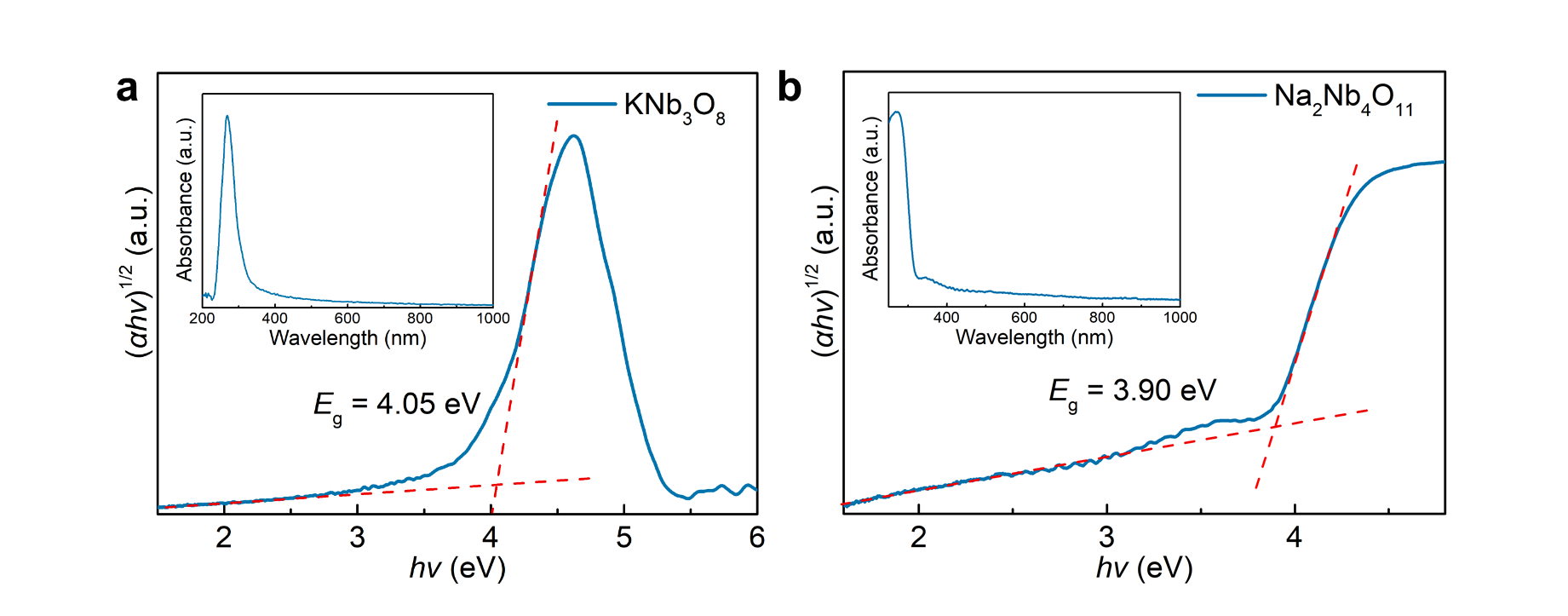


**Figure S23.** Optical bandgap identified by the micro-area UV-vis absorption spectroscopy. (a) KNO. (b) NNO. Insets: Corresponding absorption spectra.


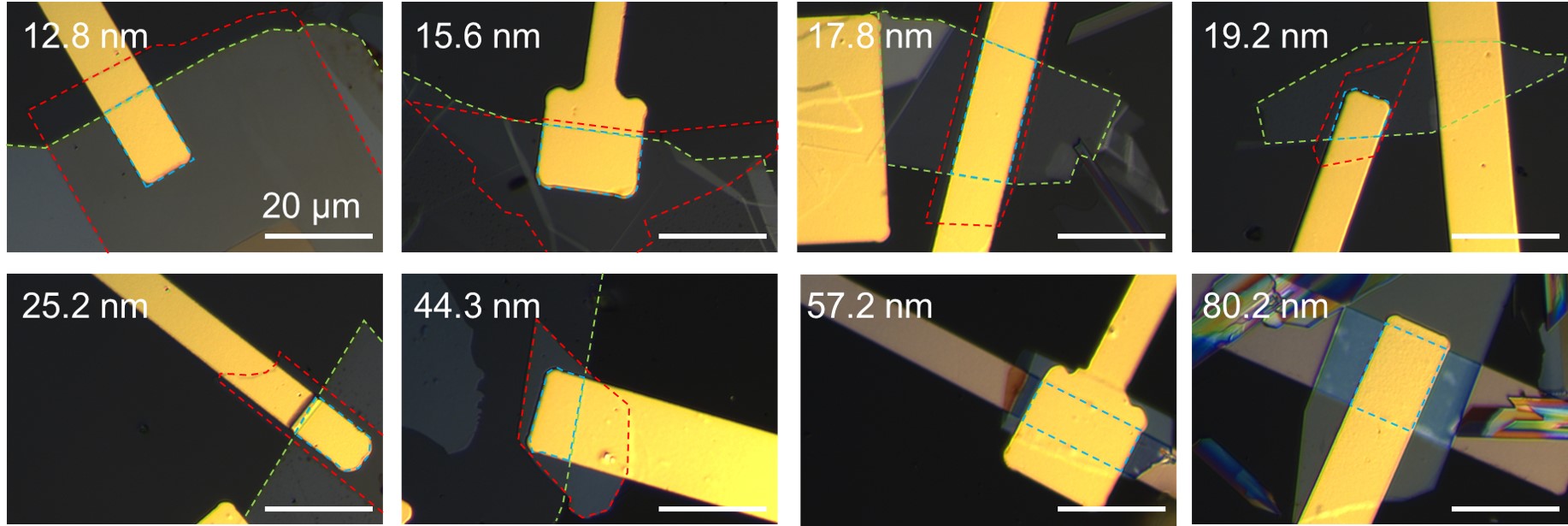


**Figure S24.** Optical images of the CNO MIM devices with varying thicknesses. The red, green, and blue dotted lines indicate the CNO nanoflakes, bottom electrodes, and overlapping area for measurements, respectively. The scale bars are all 20 μm.


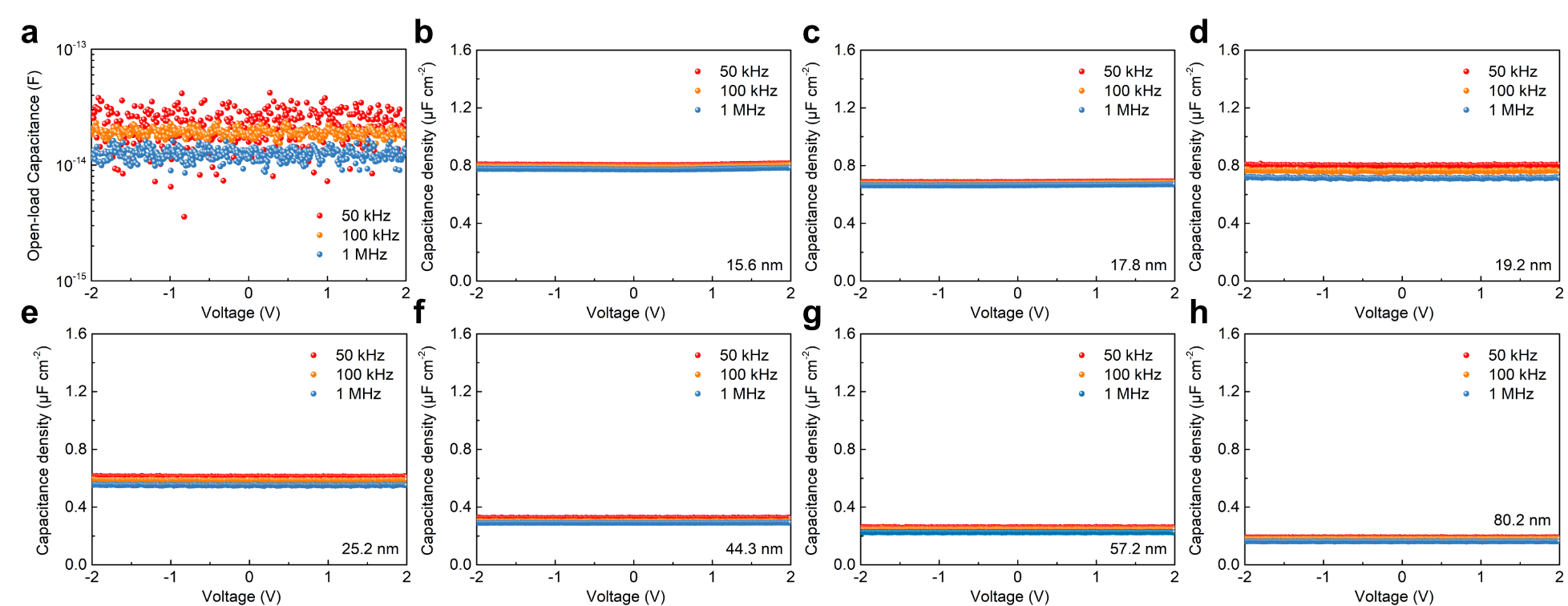


**Figure S25.** *C*-*V* characterization of CNO-based MIM devices. (a) Open-circuit load measurement showing negligible capacitance (less than 5×10^-13^ F). (b-h) Frequency-dependent *C*-*V* curves.


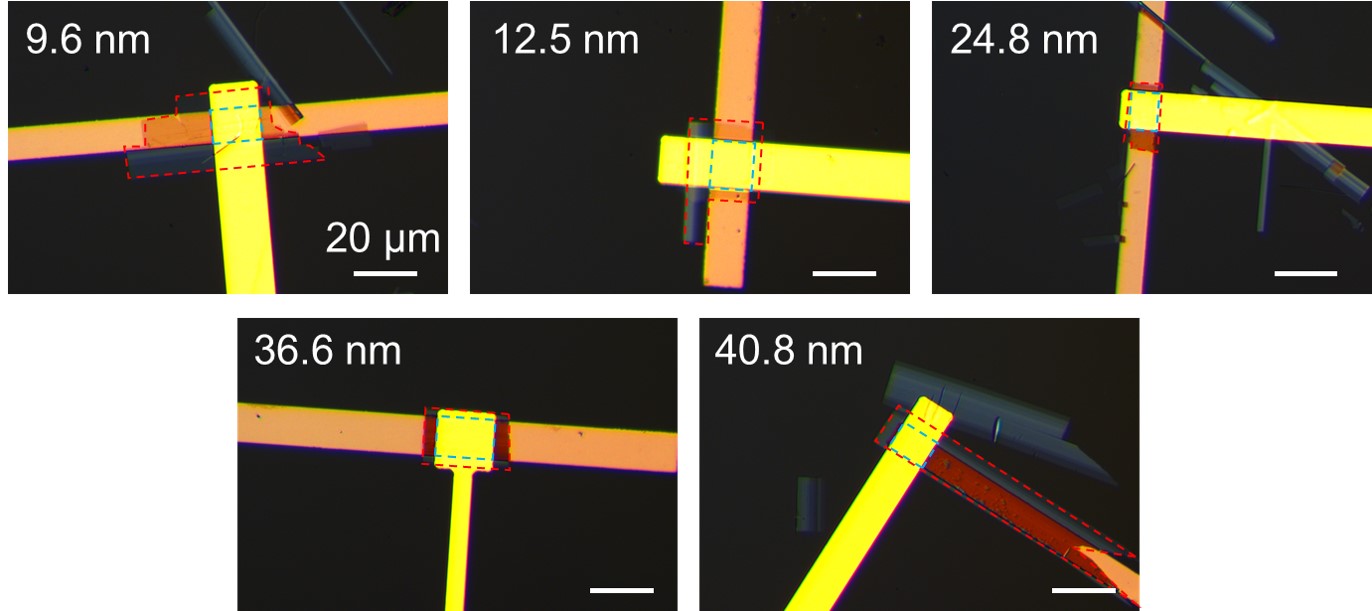


**Figure S26.** Optical images of the KNO MIM devices. The red and blue dotted lines indicate the KNO nanoflakes and overlapping area for measurements, respectively. The scale bars are all 20 μm.


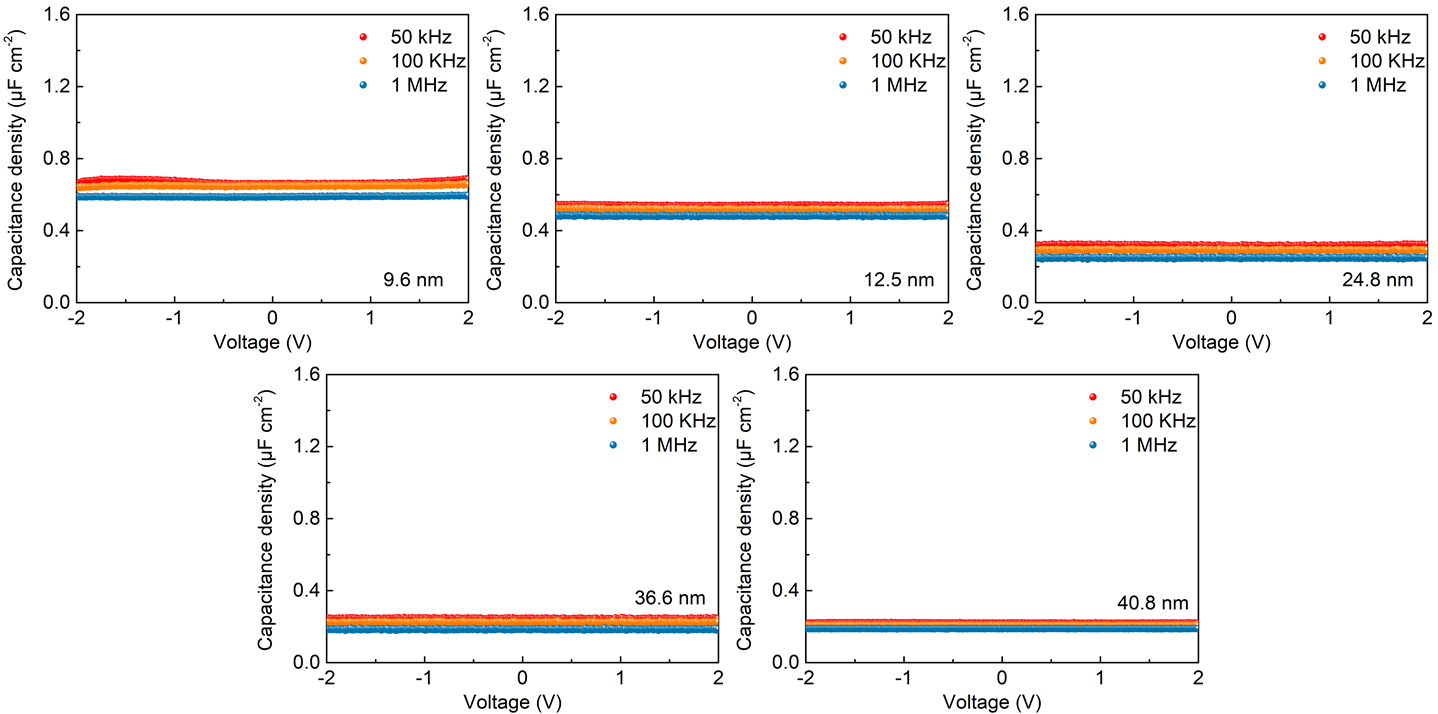


**Figure S27.** *C*-*V* curves of KNO MIM devices.


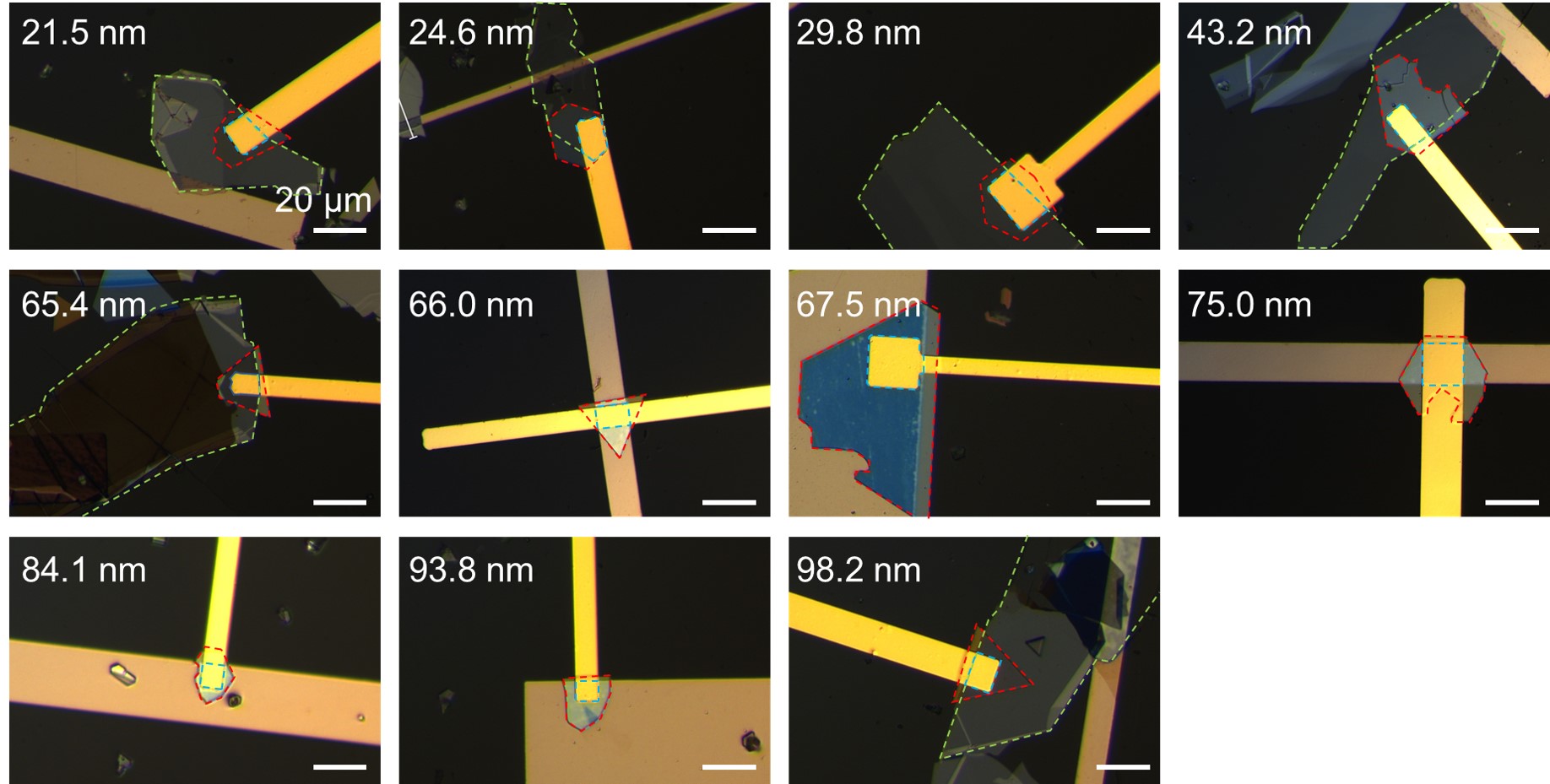


**Figure S28.** Optical images of the NNO MIM devices. The red, green, and blue dotted lines indicate the NNO nanoflakes, bottom electrodes, and overlapping area for measurements, respectively. The scale bars are all 20 μm.


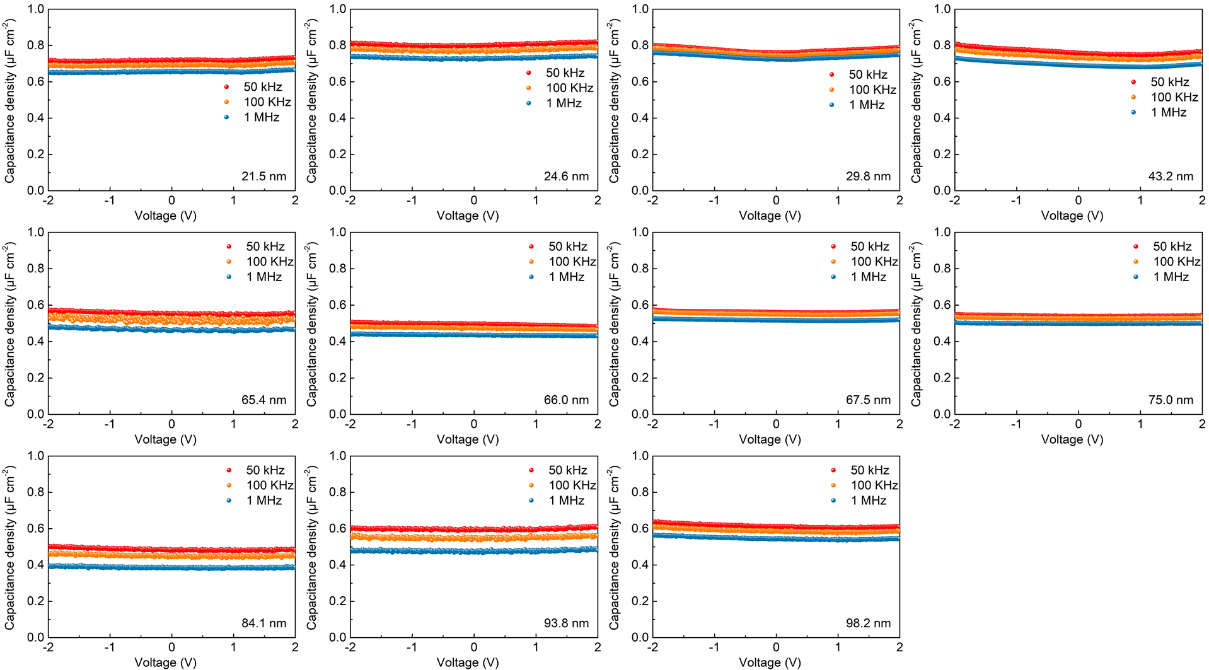


**Figure S29.** *C*-*V* curves of NNO MIM devices.

**
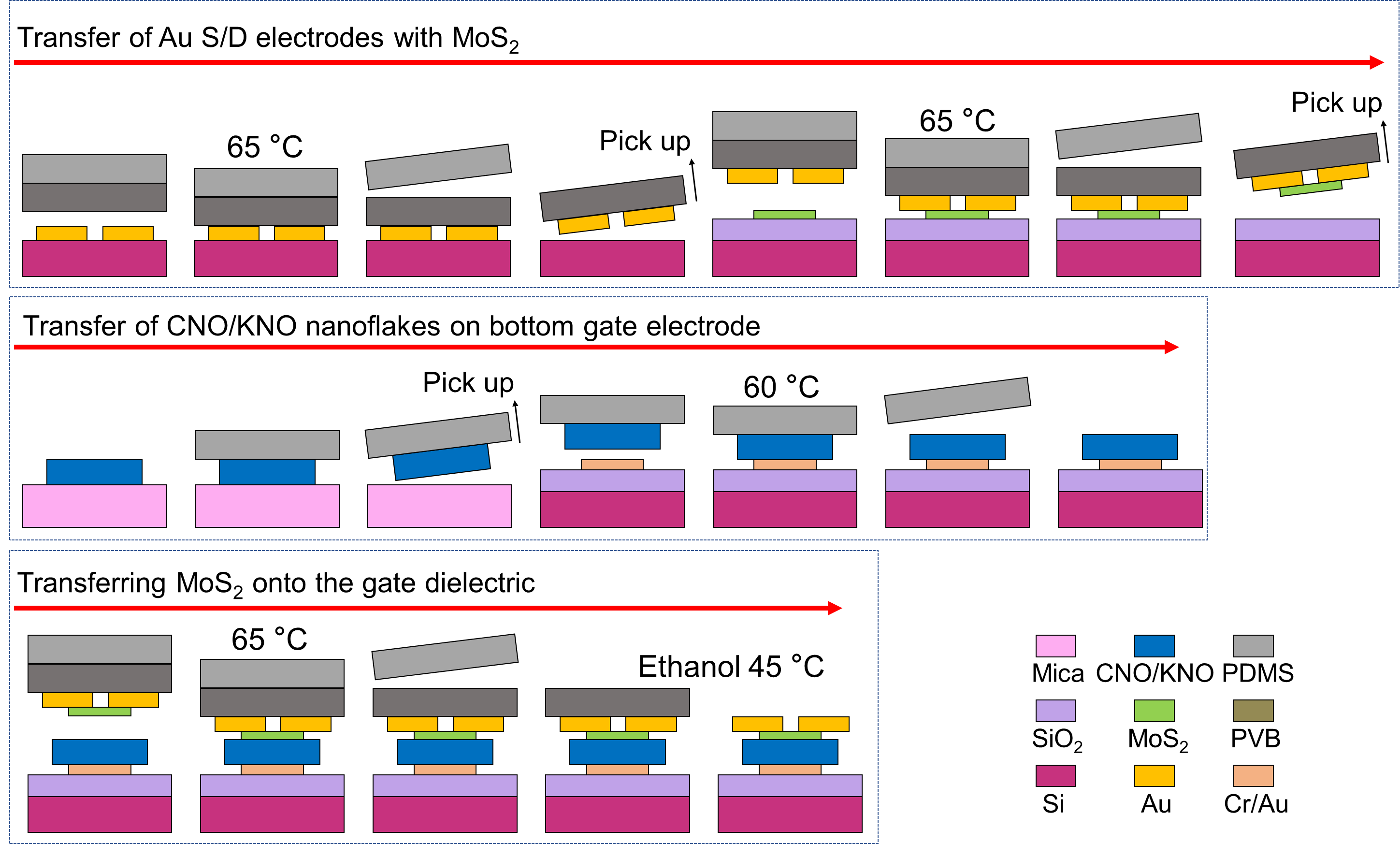
**

**Figure S30.** Dry transfer process schematic for bottom-gate FET fabrication.

**Supplementary Note III. Analysis of field effect mobility of the MoS_2_ FET with a CNO dielectric.**

The field-effect mobility (*μ*) can be obtained by the following formula:

where *L* is the channel length, *W* is the channel width, *V*_D_ is the driving voltage, *d* is the thickness of the dielectrics, *ε*_0_ is vacuum dielectric constant (8.85 × 10^-12^ F m^-1^), *ε*_r_ is the dielectric constant of the dielectrics. According to the formula, the calculated mobilities of the back-gated device using CNO nanoflakes is 24.4 cm^2^ V^-1^ s^-1^. The lower mobility may be attributed to the contact resistance between the electrodes and MoS_2_, which results in a reduced *I*_DS_ and consequently a lower measured transconductance (*dI*_DS_/*dV*_GS_).


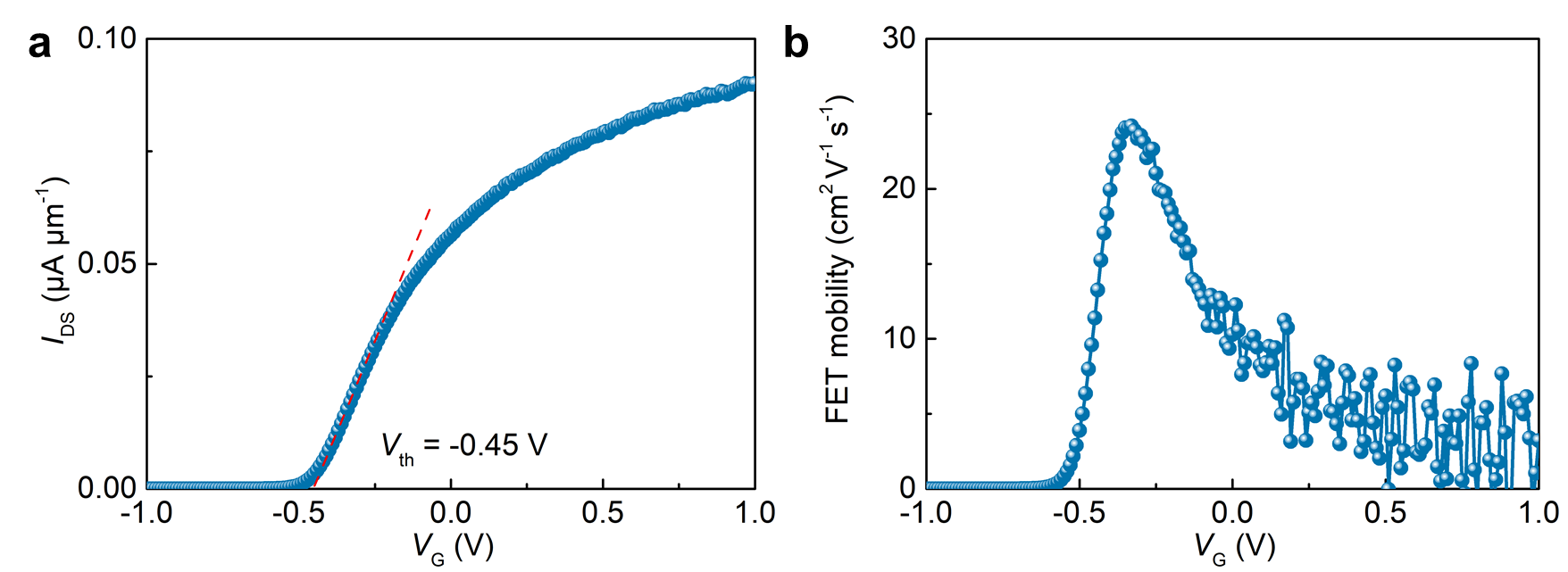


**Figure S31.** Field-effect mobility of the MoS_2_ FET with a CNO dielectric. (a) Transfer characteristics of the MoS_2_ FET on a linear scale at *V*_DS_ = 0.075 V, indicating a threshold voltage (*V*_th_) of -0.45 V. (b) Field-effect mobility as a function of gate voltage.


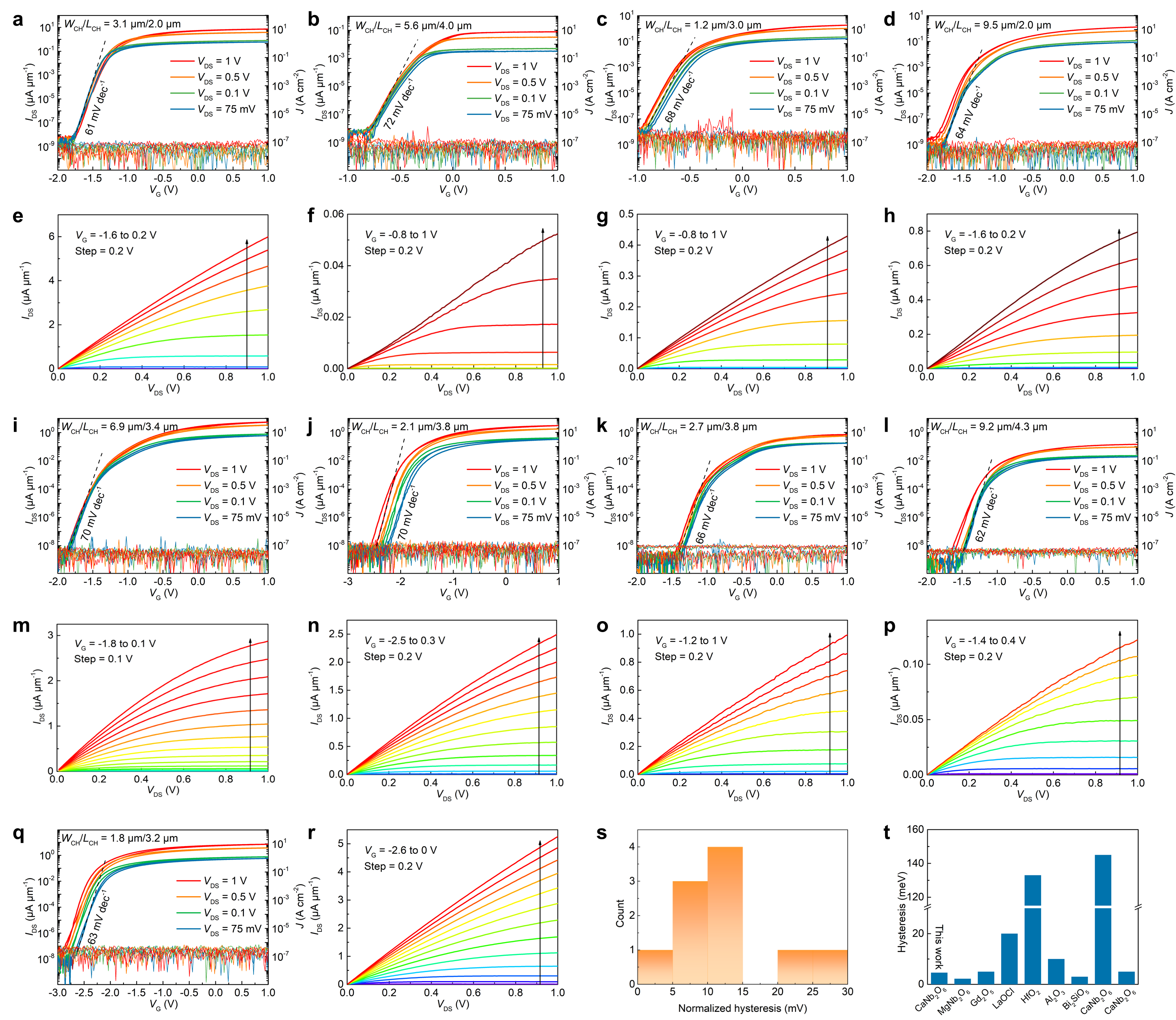


**Figure S32.** (a-r) Electrical characterization of 9 additional bottom-gate FETs with CNO gate dielectrics. (s) Hysteresis statistics of 10 bottom-gate FETs with CNO gate dielectrics at *V*_DS_ = 0.1 V in the subthreshold region (*I*_DS_ = 10^-6^ μA μm^-1^). (t) Comparison of the hysteresis for MoS_2_ transistors with high-κ dielectrics.^[4-10]^ The values for the last two CaNb_2_O_6_ devices correspond to the pre- and post-surface treatment conditions, respectively.^[10]^


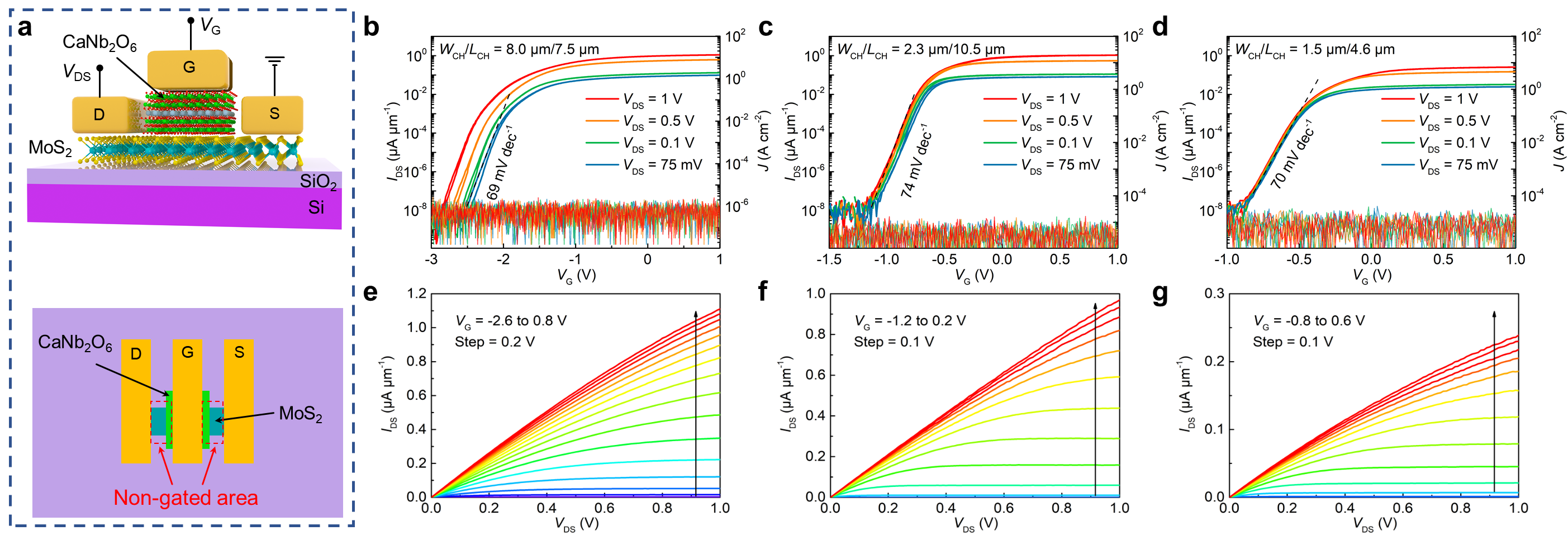


**Figure S33.** Electrical characterization of top-gate MoS_2_ FETs with CNO dielectrics. (a) Device schematic of top-gate MoS_2_ FET, with ungated regions highlighted by dashed-line box in the lower panel. (b-d) Transfer curves of top-gate devices. (e-g) Corresponding output characteristics.


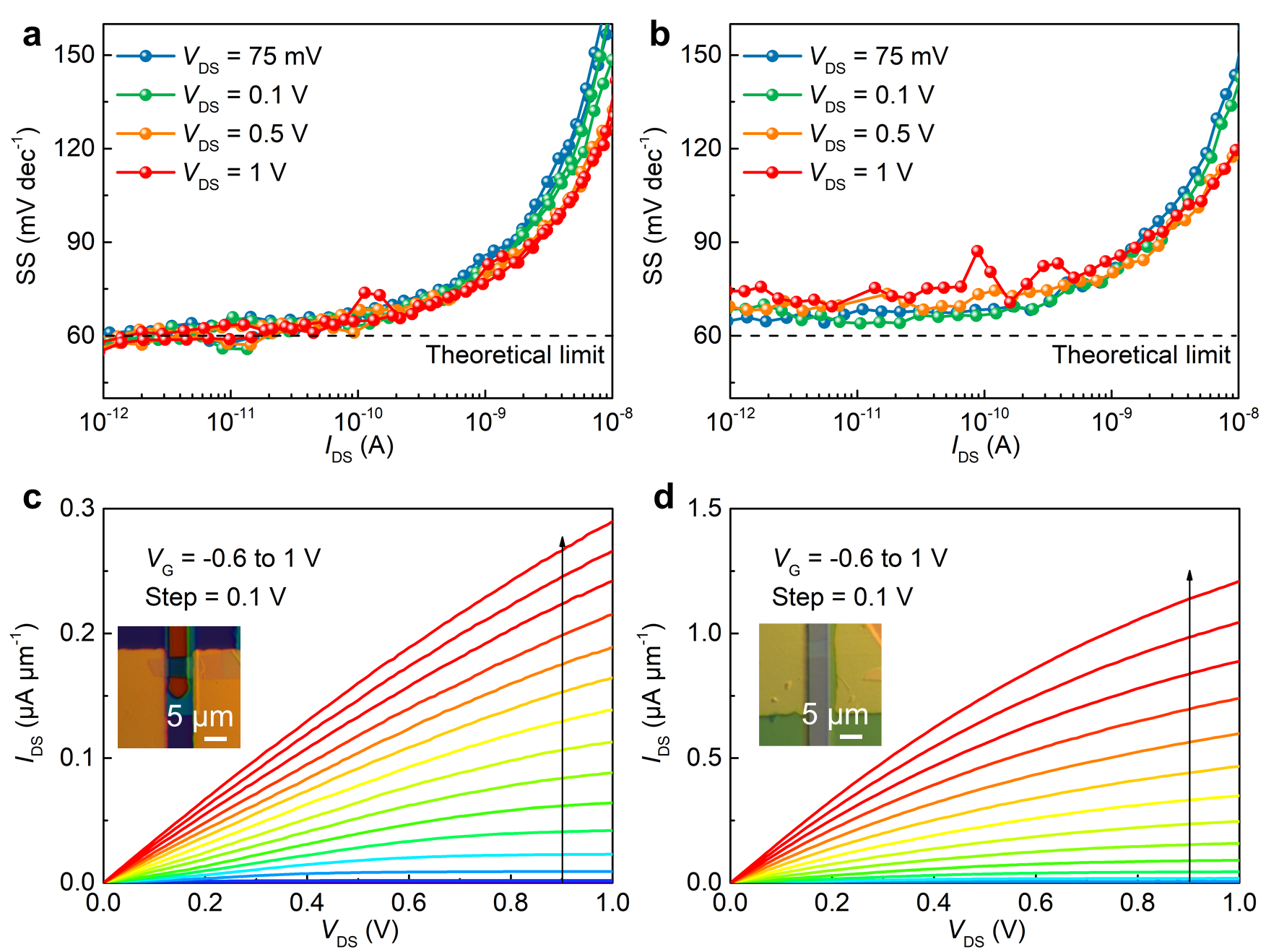


**Figure S34.** Extracted SS values from the transfer characteristics and output characteristics of bottom-gate FETs with 2D dielectric nanoflakes. (a,b) Extracted SS values versus drain current characteristics of the KNO-dielectric FET (a) and NNO-dielectric FET (b). (c) Output curves of KNO-dielectric FET. (d) Output curves of NNO-dielectric FET. The insets in (c) and (d) display the corresponding optical images of the devices.


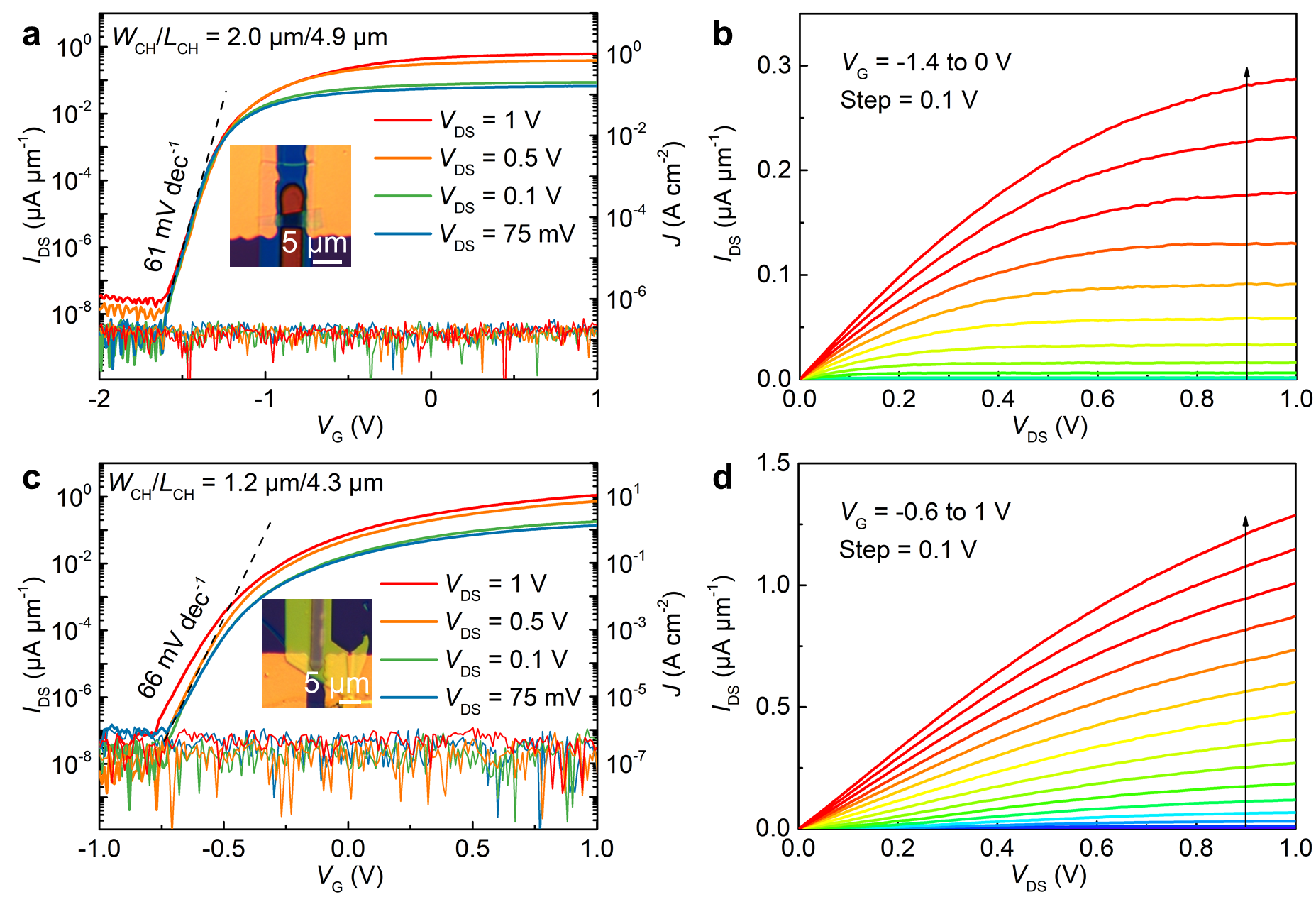


**Figure S35.** Electrical characterization of an additional device for each of KNO and NNO as bottom-gate dielectrics. (a) Transfer characteristics of KNO-gated FET showing SS of ~61 mV dec^-1^ and *I*_on_/*I*_off_ of over 10^7^. Inset: The optical images of the device. (b) Corresponding output curves. (c) NNO-gated FET transfer curves with SS of ~66 mV dec^-1^ and *I*_on_/*I*_off_ of over 10^7^. Inset: The optical images of the device. (d) Corresponding output characteristics.

**
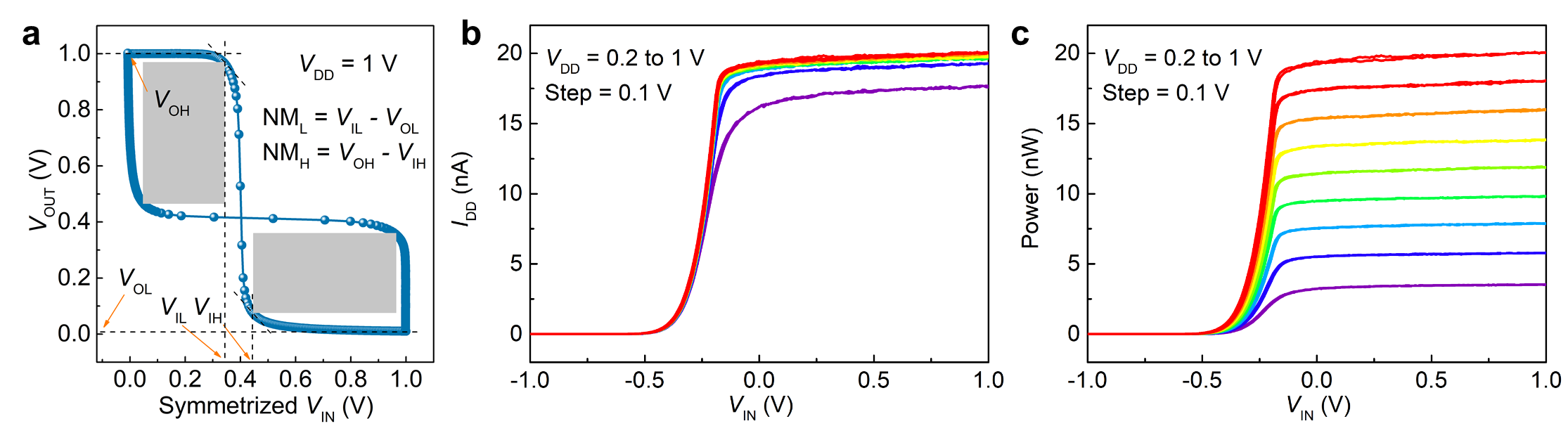
**

**Figure S36.** (a) Voltage transfer characteristics of the inverter at *V*_DD_ = 1 V, along with their mirror reflection. The *V*_IN_ axis was shifted accordingly to make it symmetric with the *V*_OUT_ axis. The key parameters *V*_OH_, *V*_OL_, *V*_IL_, *V*_IH_ denote the output high voltage, output low voltage, input low voltage, and input high voltage, respectively. The dashed lines serve as auxiliary guides for extracting the values of *V*_OH_, *V*_OL_, *V*_IL_, *V*_IH_. The NM_L_ and NM_H_ were calculated as 0.34 and 0.55, respectively. The total noise margin (NM) is calculated by (NM_L_ + NM_H_)/*V*_DD_. (b-c) The operating current (b) and corresponding static power consumption (c) of the inverter device.

**
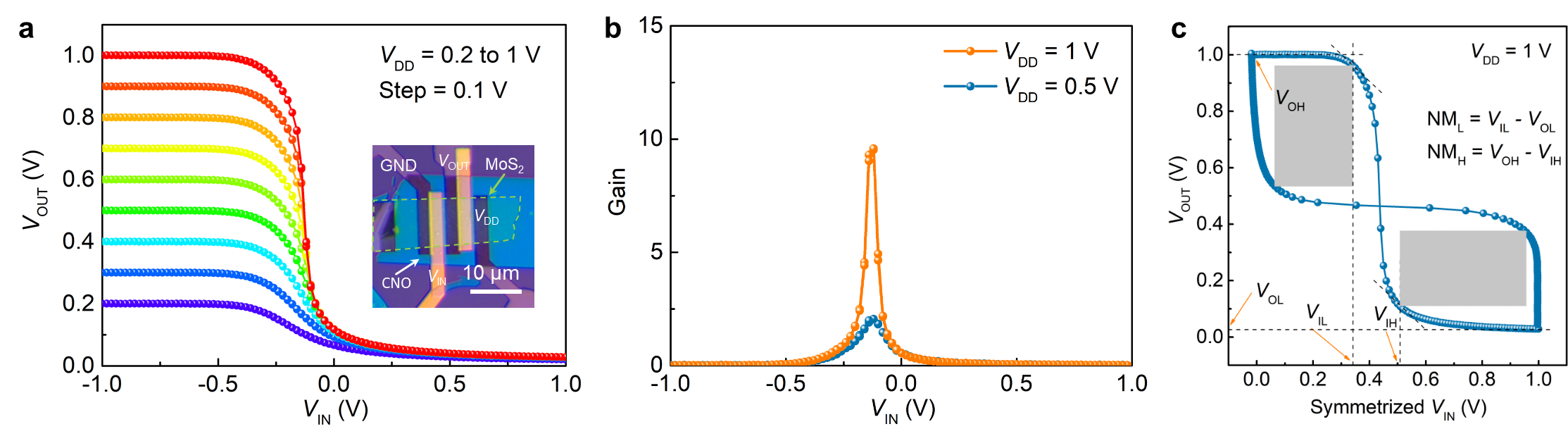
**

**Figure S37.** (a) Voltage transfer characteristics of an additional inverter, with inset showing the optical image of the device. (b) Derived voltage gain characteristics for the inverter, showing a maximum voltage gain of ~10 at *V*_DD_ = 1 V. (c) Voltage transfer characteristics of the inverter at *V*_DD_ = 1 V, along with their mirror reflection. The total noise margin is 84%.

**
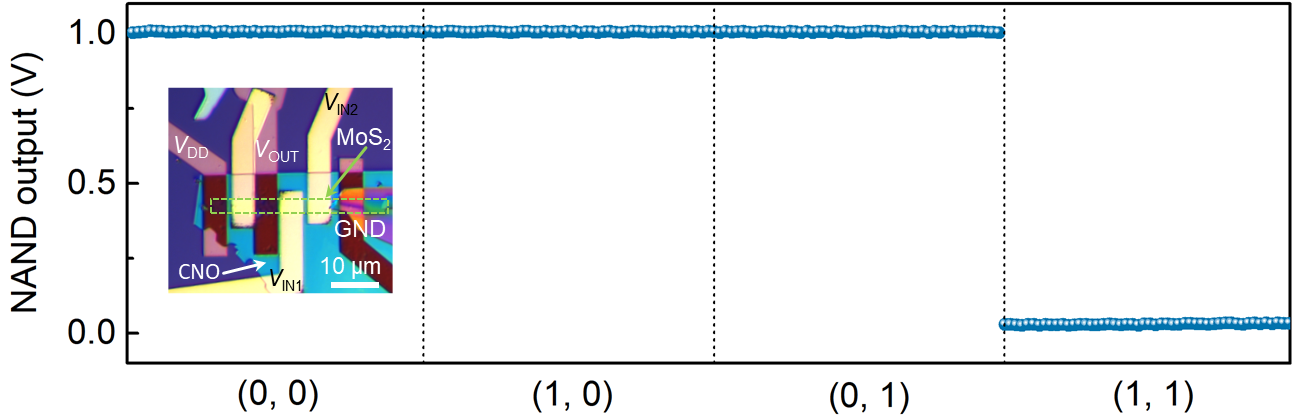
**

**Figure S38.** Input-output logic functionality of an additional NAND gate at *V*_DD_ = 1 V. The inset shows the optical image of the device.

**Table S1** Comparison of dielectric properties of various materials.

| Dielectric | Bandgap (eV) | Dielectric constant (*ε*_r_) | Breakdown strength (MV cm^-1^) | References |
| --- | --- | --- | --- | --- |
| SiO_2_ | 9 | 3.9 | 40 | [11-13] |
| HfO_2_ | 5.25 | 22 | 4.9-8.5 | [11, 14] |
| Al_2_O_3_ | 6.95 | 7.4 | 6 | [11, 15] |
| CaF_2_ | 12.1 | 8.4 | 5-27.8 | [16, 17] |
| Sb_2_O_3_ | 3.95 | 11.5 | 2.7 | [18] |
| LaOCl | 5.54 | 10.8-13.7 | 10 | [6, 19] |
| GdOCl | 4.58 | 15.3 | 9.9 | [20] |
| SrTiO_3_ | 3.3 | 20-100 | 2.5-6.0 | [21-23] |
| Bi_2_SeO_5_ | 3.6 | 16.5 | 10 | [24] |
| Bi_2_SiO_5_ | 3.8 | 32 | 9.4 | [9] |
| *h*-BN | 5.2 | 2-4 | 2.5-7.9 | [25-28] |
| MgNb_2_O_6_ | 5.0 | 20 | 16 | [4] |
| CaNb_2_O_6_ | 4.17 | 16 | 12 | [10] |
| **CaNb_2_O_6_** | **4.32** | **16** | **15.7** | **This work** |
| **KNb_3_O_8_** | **3.98** | **9** | **5.8** | **This work** |
| **Na_2_Nb_4_O_11_** | **4.12** | **68** | **4.9** | **This work** |

**Table S2** A comparison of the FETs with state-of-the-art dielectric layers for MoS_2_ transistors.

| Dielectric | SS  (mV dec^-1^) | *I*_on_/*I*_off_ | *D*_it_ (cm^-2^ eV^-1^) | Reference |
| --- | --- | --- | --- | --- |
| HfO_2_ | 126 | 10^7^ | 3.02×10^12^ | [7] |
| HfO_2_ | 74 | 10^8^ | n/a | [29] |
| Al_2_O_3_ | 68 | 10^7^ | 7.6×10^9^ | [15] |
| CaF_2_ | 90 | 10^7^ | 1×10^13^ | [30] |
| HfO_2_/PTCDA | 60 | 10^7^ | 8×10^11^ | [31] |
| hBN | 80 | 10^6^ | n/a | [32] |
| Gd_2_O_5_ | 61.5 | 10^8^ | 2.2×10^12^ | [5] |
| Er_2_O_3_ | 88 | 10^6^ | 6×10^11^ | [33] |
| Sb_2_O_3_ | 60 | 10^7^ | 2.2×10^11^ | [34] |
| Sb_2_O_3_ | 64 | 10^8^ | n/a | [18] |
| GdOCl | 85 | 10^9^ | 1.07×10^11^ | [35] |
| GdOCl | 67.9 | 10^6^ | 7.81×10^11^ | [20] |
| LaOCl | 77.1 | 1×10^7^ | n/a | [36] |
| LaOCl | 80 | 2×10^8^ | 1.56×10^12^ | [6] |
| LaOCl | 147.2 | 10^4^ | n/a | [19] |
| SrTiO_3_ | 71.5 | 10^7^ | 4.3×10^12^ | [22] |
| SrTiO_3_ | 66 | 10^8^ | n/a | [21] |
| Bi_2_SeO_5_ | 70 | 10^8^ | n/a | [24] |
| MgNb_2_O_6_ | 62 | 4×10^7^ | 4×10^11^ | [4] |
| CaNb_2_O_6_ | 145 | 10^7^ | n/a | [10] |
| CaNb_2_O_6_ (air-plasma) | 61 | 10^7^ | 1.4 × 10^11^ | [10] |
| **CaNb_2_O_6_** | **60** | **4×10^7^** | **2.2×10^10^** | **This work** |
| **KNb_3_O_8_** | **60** | **2×10^7^** | **9.4×10^9^** | **This work** |
| **Na_2_Nb_4_O_11_** | **65** | **9×10^7^** | **2.7×10^11^** | **This work** |

**References**

[1] U. Duman, M. Aycibin, Ö. F. Özdemir, *Phys. Status Solidi (b)* **2021**, *258*, 2100416.

[2] Y. Ping, H. Long, H. Liu, C. Chen, N. Zhang, H. Jing, J. Lu, Y. Zhao, Z. Yang, W. Li, F. Ma, X. Fang, Z. Wei, H. Xu, *Adv. Funct. Mater.* **2022**, *32*, 2111673.

[3] J.-K. Qin, H. Xiao, C.-Y. Zhu, L. Zhen, C.-Y. Xu, *Adv Opt Mater* **2022**, *10*, 2201627.

[4] C.-Y. Zhu, M.-R. Zhang, Q. Chen, L.-Q. Yue, R. Song, C. Wang, H.-Z. Li, F. Zhou, Y. Li, W. Zhao, L. Zhen, M. Si, J. Li, J. Wang, Y. Chai, C.-Y. Xu, J.-K. Qin, *Nat. Electron.* **2024**, *7*, 1137.

[5] L. Yin, R. Cheng, X. Wan, J. Ding, J. Jia, Y. Wen, X. Liu, Y. Guo, J. He, *Nat. Mater.* **2025**, *24*, 197.

[6] Z. Fu, C. Jian, Y. Yao, Y. Li, J. Yuan, Q. Cai, W. Liu, *Adv. Funct. Mater.* **2025**, *35*, 2501136.

[7] M. Wen, J. Xu, L. Liu, P.-T. Lai, W.-M. Tang, *Appl. Phys. Express* **2016**, *9*, 095202.

[8] T. Li, B. Wan, G. Du, B. Zhang, Z. Zeng, *AIP Adv.* **2015**, *5*, 057102.

[9] J. Chen, Z. Liu, X. Dong, Z. Gao, Y. Lin, Y. He, Y. Duan, T. Cheng, Z. Zhou, H. Fu, F. Luo, J. Wu, *Nat. Commun.* **2023**, *14*, 4406.

[10] X. Fan, J. Yi, B. Deng, C. Zhou, Z. Zhang, J. Yu, W. Li, C. Li, G. Wu, X. Zhou, T. Sun, Y. Zhu, J. Zhou, J. Xia, Z. Wang, K. Lai, Z. Peng, D. Li, A. Pan, Y. Zhou, *Nat. Commun.* **2025**, *16*, 2585.

[11] E. Bersch, S. Rangan, R. A. Bartynski, E. Garfunkel, E. Vescovo, *Phys. Rev. B* **2008**, *78*, 085114.

[12] C. Sire, S. Blonkowski, M. J. Gordon, T. Baron, *Appl. Phys. Lett.* **2007**, *91*, 242905.

[13] J. Robertson, *The European Physical Journal - Applied Physics* **2004**, *28*, 265.

[14] K. Laegu, L. Byoung Hun, Q. Wen-Jie, J. Yongjoo, R. Nieh, S. Gopalan, K. Onishi, J. C. Lee, *IEEE Electron Device Letters* **2000**, *21*, 181.

[15] Z. Lu, Y. Chen, W. Dang, L. Kong, Q. Tao, L. Ma, D. Lu, L. Liu, W. Li, Z. Li, X. Liu, Y. Wang, X. Duan, L. Liao, Y. Liu, *Nat. Commun.* **2023**, *14*, 2340.

[16] C. Wen, A. G. Banshchikov, Y. Y. Illarionov, W. Frammelsberger, T. Knobloch, F. Hui, N. S. Sokolov, T. Grasser, M. Lanza, *Adv. Mater.* **2020**, *32*, 2002525.

[17] N. S. Sokolov, I. V. Grekhov, S. Ikeda, A. K. Kaveev, A. V. Krupin, K. Saiki, K. Tsutsui, S. E. Tyaginov, M. I. Vexler, *Microelectron. Eng.* **2007**, *84*, 2247.

[18] K. Liu, B. Jin, W. Han, X. Chen, P. Gong, L. Huang, Y. Zhao, L. Li, S. Yang, X. Hu, J. Duan, L. Liu, F. Wang, F. Zhuge, T. Zhai, *Nat. Electron.* **2021**, *4*, 906.

[19] L. Li, W. Dang, X. Zhu, H. Lan, Y. Ding, Z.-A. Li, L. Wang, Y. Yang, L. Fu, F. Miao, M. Zeng, *Adv. Mater.* **2023**, *37*, 2309296.

[20] W. Xu, J. Jiang, Y. Chen, N. Tang, C. Jiang, S. Yang, *Nat. Commun.* **2024**, *15*, 9469.

[21] A. J. Yang, K. Han, K. Huang, C. Ye, W. Wen, R. Zhu, R. Zhu, J. Xu, T. Yu, P. Gao, Q. Xiong, X. Renshaw Wang, *Nat. Electron.* **2022**, *5*, 233.

[22] J.-K. Huang, Y. Wan, J. Shi, J. Zhang, Z. Wang, W. Wang, N. Yang, Y. Liu, C.-H. Lin, X. Guan, L. Hu, Z.-L. Yang, B.-C. Huang, Y.-P. Chiu, J. Yang, V. Tung, D. Wang, K. Kalantar-Zadeh, T. Wu, X. Zu, L. Qiao, L.-J. Li, S. Li, *Nature* **2022**, *605*, 262.

[23] K. van Benthem, C. Elsässer, R. H. French, *J. Appl. Phys.* **2001**, *90*, 6156.

[24] C. Zhang, T. Tu, J. Wang, Y. Zhu, C. Tan, L. Chen, M. Wu, R. Zhu, Y. Liu, H. Fu, J. Yu, Y. Zhang, X. Cong, X. Zhou, J. Zhao, T. Li, Z. Liao, X. Wu, K. Lai, B. Yan, P. Gao, Q. Huang, H. Xu, H. Hu, H. Liu, J. Yin, H. Peng, *Nat. Mater.* **2023**, *22*, 832.

[25] K. K. Kim, A. Hsu, X. Jia, S. M. Kim, Y. Shi, M. Dresselhaus, T. Palacios, J. Kong, *ACS Nano* **2012**, *6*, 8583.

[26] S. M. Kim, A. Hsu, M. H. Park, S. H. Chae, S. J. Yun, J. S. Lee, D.-H. Cho, W. Fang, C. Lee, T. Palacios, M. Dresselhaus, K. K. Kim, Y. H. Lee, J. Kong, *Nat. Commun.* **2015**, *6*, 8662.

[27] G.-H. Lee, Y.-J. Yu, C. Lee, C. Dean, K. L. Shepard, P. Kim, J. Hone, *Appl. Phys. Lett.* **2011**, *99*, 243114.

[28] D. M. Hoffman, G. L. Doll, P. C. Eklund, *Phys. Rev. B* **1984**, *30*, 6051.

[29] B. Radisavljevic, A. Radenovic, J. Brivio, V. Giacometti, A. Kis, *Nat. Nanotechnol.* **2011**, *6*, 147.

[30] Y. Y. Illarionov, A. G. Banshchikov, D. K. Polyushkin, S. Wachter, T. Knobloch, M. Thesberg, L. Mennel, M. Paur, M. Stöger-Pollach, A. Steiger-Thirsfeld, M. I. Vexler, M. Waltl, N. S. Sokolov, T. Mueller, T. Grasser, *Nat. Electron.* **2019**, *2*, 230.

[31] W. Li, J. Zhou, S. Cai, Z. Yu, J. Zhang, N. Fang, T. Li, Y. Wu, T. Chen, X. Xie, H. Ma, K. Yan, N. Dai, X. Wu, H. Zhao, Z. Wang, D. He, L. Pan, Y. Shi, P. Wang, W. Chen, K. Nagashio, X. Duan, X. Wang, *Nat. Electron.* **2019**, *2*, 563.

[32] G.-H. Lee, X. Cui, Y. D. Kim, G. Arefe, X. Zhang, C.-H. Lee, F. Ye, K. Watanabe, T. Taniguchi, P. Kim, J. Hone, *ACS Nano* **2015**, *9*, 7019.

[33] H. Uchiyama, K. Maruyama, E. Chen, T. Nishimura, K. Nagashio, *Small* **2023**, *19*, 2207394.

[34] Y. Xu, T. Liu, K. Liu, Y. Zhao, L. Liu, P. Li, A. Nie, L. Liu, J. Yu, X. Feng, F. Zhuge, H. Li, X. Wang, T. Zhai, *Nat. Mater.* **2023**, *22*, 1078.

[35] Z. Liu, L. Yin, X. Peng, Y. Zhou, B. Zhai, Y. Yu, R. Cheng, Y. Wen, J. Jiang, X. Feng, F. Wang, J. He, *Adv. Mater.* **2025**, *37*, 2417103.

[36] B. Zhang, Y. Zhu, Y. Zeng, Z. Zhao, X. Huang, D. Qiu, Z. Fang, J. Wang, J. Xu, R. Wang, S. Gao, Y. Hou, *J. Am. Chem. Soc.* **2023**, *145*, 11074.
